# Control of Polarity in Kagome‐NiAs Bismuthides

**DOI:** 10.1002/anie.202403670

**Published:** 2024-04-03

**Authors:** Quinn D. Gibson, Dongsheng Wen, Hai Lin, Marco Zanella, Luke M. Daniels, Craig M. Robertson, John B. Claridge, Jonathan Alaria, Matthew S. Dyer, Matthew J. Rosseinsky

**Affiliations:** ^1^ Department of Chemistry University of Liverpool Crown Street Liverpool L69 7ZD United Kingdom; ^2^ Present Address for Quinn D. Gibson Aberdeen Centre for Energy and Sustainability Department of Chemistry University of Aberdeen Aberdeen AB24, 3FX United Kingdom; ^3^ Department of Physics University of Liverpool Oliver Lodge Laboratory Liverpool L69 7ZE United Kingdom

**Keywords:** Kagome, NiAs, Intermetallic, Polar metal

## Abstract

A 2×2×1 superstructure of the *P*6_3_/*mmc* NiAs structure is reported in which kagome nets are stabilized in the octahedral transition metal layers of the compounds Ni_0.7_Pd_0.2_Bi, Ni_0.6_Pt_0.4_Bi, and Mn_0.99_Pd_0.01_Bi. The ordered vacancies that yield the true hexagonal kagome motif lead to filling of trigonal bipyramidal interstitial sites with the transition metal in this family of “kagome‐NiAs” type materials. Further ordering of vacancies within these interstitial layers can be compositionally driven to simultaneously yield kagome‐connected layers and a net polarization along the *c* axes in Ni_0.9_Bi and Ni_0.79_Pd_0.08_Bi, which adopt *Fmm*2 symmetry. The polar and non‐polar materials exhibit different electronic transport behaviour, reflecting the tuneability of both structure and properties within the NiAs‐type bismuthide materials family.

## Graphical abstract

Ordered octahedral site vacancies and filling of trigonal bipyramidal interstitial sites by transition metals stabilizes a 2×2×1 *P*6_3_/*mmc* superstructure of NiAs with kagome nets in Ni_0.7_Pd_0.2_Bi, Ni_0.6_Pt_0.4_Bi, and Mn_0.99_Pd_0.01_Bi. Further ordering of the interstitial sites via composition generates a net polarization in Ni_0.9_Bi and Ni_0.79_Pd_0.08_Bi with polar *Fmm*2 symmetry. The control of polarity is reflected in electronic transport properties.

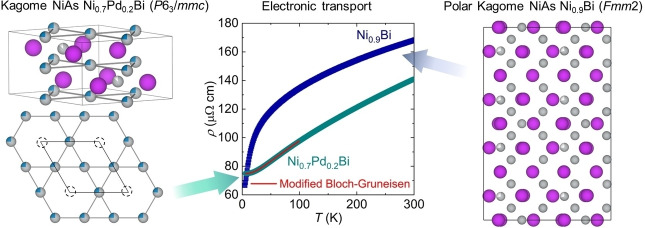

## Introduction

**Figure 1:**
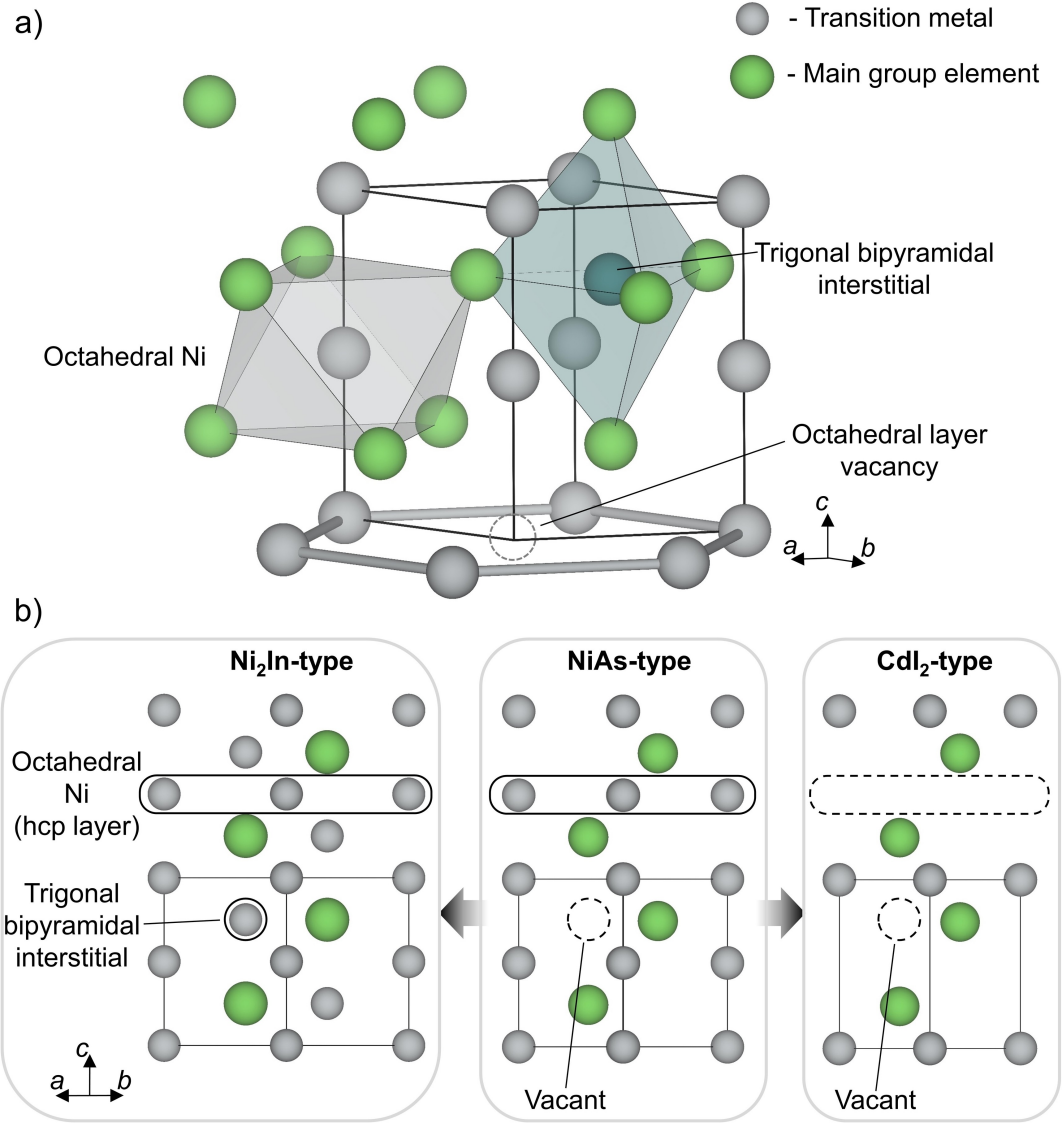
a) NiAs structure formed from layers of transition metal (grey) octahedrally coordinated by a main group element (green) forming a hexagonal lattice. Possible defects in the NiAs structure include transition metal occupancy of interstitial sites in trigonal bipyramidal voids (blue), or vacancies within the octahedral transition metal layer (dashed grey circle). b) Structural evolution of the NiAs structure type (middle). The structure is Ni_2_In‐type (left) when the trigonal bipyramidal interstitial sites are fully occupied (represented by solid black outlines). The structure is CdI_2_‐type (right) when every other layer of octahedral sites is removed through ordering of transition metal vacancies (empty sites represented by dashed outlines). A range of transition metal to main group metal ratios are possible, and can lead to diverse superstructures. Atom colours: transition metal—grey, main group element—green.

The NiAs structure type, also known as the B8 structure, is one of the most well‐known intermetallic structure types in solid state chemistry.^[1]^ In this structure, the main group element produces a hexagonal closed packed lattice, and the transition metal atom occupies all of the octahedral voids, thus also forming hcp layers (Figure 1a). The NiAs structure has been observed for materials containing 3d, 4d, and 5d transition metals, and main group elements ranging from phosphorous to bismuth.

Numerous superstructures of the NiAs structure exist, and can be generated through the filling of interstitial trigonal bipyramidal holes with transition metals, or through the ordering of metal vacancies in the octahedral voids of the hcp array (Figure 1). When the interstitial trigonal bipyramidal sites are completely filled, the structure is Ni_2_In‐type (Figure 1b). As intermetallics do not require formal charge balance, materials with a wide range of ratios of transition metal to main group element between 1 : 1 and 2 : 1 can be synthesized with different interstitial orderings, ranging from fully disordered to incommensurately ordered.[^2^, ^3^, ^4^, ^5^, ^6^] Ordered vacancies within the transition metal layer of NiAs can yield ratios less than 1 : 1, such as for Fe_7_S_8_,^[7]^ all the way down to the 1 : 2 ratio which yields the CdI_2_ structure (Figure 1b).^[8]^ Further superstructures can be made wherein there is mixing between the transition metal and main group sites.^[6]^ There are also structures that do not fit neatly into the CdI_2_−NiAs−Ni_2_In series, where ordered vacancies in the octahedral layer coexist with ordered interstitials in the trigonal bipyramidal layer. These orderings across both sites can lead to complex long range order and the creation of a polar axis, such as for metastable *Fdd*2 Mn_1.05_Bi,^[9]^ which can be thermodynamically stabilised through the inclusion of Rh on the interstitial site.^[10]^ Mn_1.05_Bi and its Rh stabilized relative are the only materials known to adopt this type of structure, with Ni_1+d_Bi adopting a similar structure but with a non‐polar ordering of interstitials.^[11]^ All of these materials have at least some layers of filled octahedral sites that form a hcp (or distorted hcp) layer of transition metals.

The kagome net is an important hexagonal symmetry motif in intermetallics that consists of corner‐sharing triangles.[^12^, ^13^, ^14^] It can lead to unique band topology near the Fermi level that can result in a host of properties including superconductivity or massive Dirac fermions.[^15^, ^16^, ^17^, ^18^, ^19^, ^20^, ^21^] It is possible to form kagome nets by the ordering of 25 % vacancies of an hcp net, as found in the NiAs structure, creating the key motif of hexagons surrounded by triangles. We report the intermetallics Ni_0.7_Pd_0.2_Bi, Ni_0.6_Pt_0.4_Bi, and Mn_0.99_Pd_0.01_Bi, which adopt derivatives of the NiAs structure type, but feature true kagome nets of the octahedral transition metals realized by control of vacancy order. Two structurally related materials demonstrate that it is possible to further combine nets displaying kagome connectivity with orderings of the interstitial sites that break inversion symmetry and generate a polar axis, which can be compositionally tuned in this family of NiAs‐based superstructures.

The generation of a polar axis in metallic materials is of interest both due to electronic properties generated by the polar axis,[^22^, ^23^] as well as the analogy to insulating ferroelectric materials.[^24^, ^25^] Firstly, a polar axis, when combined with spin orbit coupling allows for spin‐splitting in the bulk electronic structure, leading to the possibility of bulk Weyl nodes. Secondly, polar ferroelectric distortions are generally suppressed by screening of the dipole by mobile charge carriers; such distortions can be still generated by considering geometric and bonding factors.^[26]^ Therefore, different design principles can be applied in metals, analogous to the difference between improper and proper ferroelectrics in insulators. As such, the ability to tune the polarity via defect ordering in the NiAs‐type structures presented here represents a method of chemically switching off and on the axis of structural polarization in a metallic system.

## Results and Discussion

**Figure 2:**
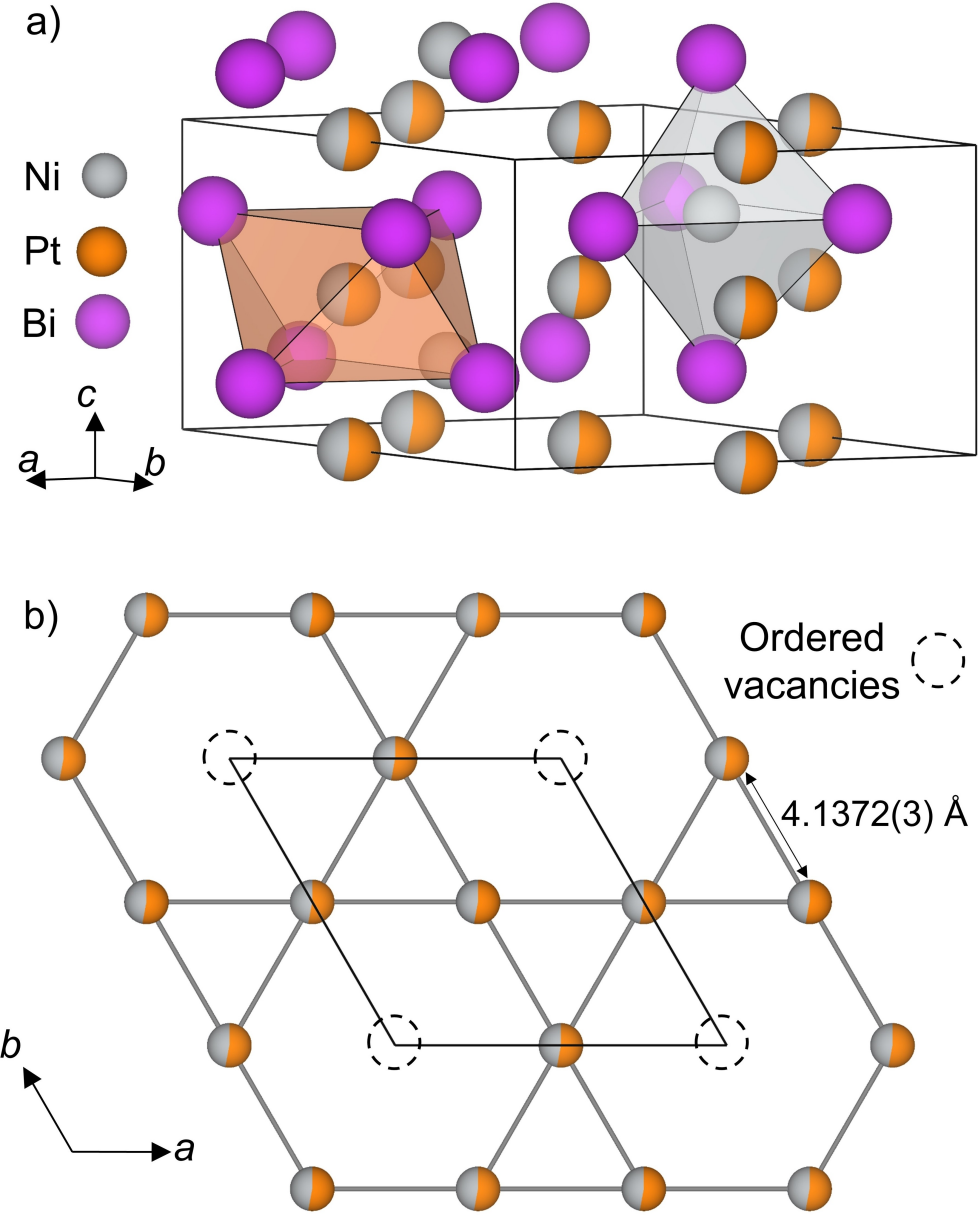
a) Crystal structure of “kagome‐NiAs” Ni_0.6_Pt_0.4_Bi. This 2×2×1 superstructure of NiAs arises from ordered removal of 25 % of the octahedrally coordinated metal, and ordered filling of 25 % of the trigonal bipyramidal sites with Ni. b) The ordering of vacancies within the octahedral layer (dashed circles) yields a true hexagonal kagome net of transition metal sites. The octahedral layer consists of mixed Pt/Ni sites (Pt occupancy: 0.530(9); Ni occupancy: 0.470(9)), and trigonal bipyramidal voids are filled with Ni (Ni occupancy: 1). Atom colours: Ni—grey, Pt—orange, Bi—purple.

Five new intermetallic materials are discovered through synthesis in the Ni−Pd‐Bi, Ni−Pt‐Bi, and Mn−Pd‐Bi phase spaces using excess Bi as a flux metal. Pd and Pt were chosen as dopant atoms into the Ni−Bi phase system as they are isolectronic to Ni, and thus electron count effects can be controlled for. Furthermore, Pd and Pt both show good solubility in molten Bi, allowing single crystal growth in a Bi rich flux. These materials are summarized in Table 1. Among them, a derivative of the NiAs structure type emerges, which is adopted by three isostructural materials, Ni_0.7_Pd_0.2_Bi, Ni_0.6_Pt_0.4_Bi, and Mn_0.99_Pd_0.01_Bi (the compositions of which come from refined single crystal data and are consistent with compositions measured using energy dispersive X‐ray spectroscopy, Figures S1–S5), which all adopt a 2×2×1 *P*6_3_/*mmc* superstructure of NiAs (Figure 2a, Tables S1–S15). This superstructure is generated by the ordering of 25 % vacancies within the octahedral layer, yielding true hexagonal kagome transition metal nets (Figure 2b), and ordered filling of 25 % of the trigonal bipyramidal sites, retaining the non‐polar *P*6_3_/*mmc* symmetry in each of the new materials. This “kagome‐NiAs” structure type is accessed via doping of NiBi and MnBi with second or third row transition metals in a range of concentrations. Indeed, only a very small amount (between 1–3 %) of Pd doping at the composition Mn_0.99_Pd_0.01_Bi leads to crystallization of the 2×2×1 kagome‐NiAs superstructure. This is distinct from undoped MnBi, which is isostructural with *P*6_3_/*mmc* NiAs. The crystal structure of Ni_0.6_Pt_0.4_Bi is shown in Figure 2a. The transition metals, Ni and Pt, are disordered across the kagome net formed from the vacancy‐ordered octahedral sites, which is the same in Ni_0.7_Pd_0.2_Bi and Mn_0.99_Pd_0.01_Bi. Ni and Mn fully occupy the trigonal bipyramidal interstitial sites in Ni_0.6_Pt_0.4_Bi and Mn_0.99_Pd_0.01_Bi, respectively, with a Ni occupancy of 0.864(14) in Ni_0.7_Pd_0.2_Bi.

**Table 1:** Summary of the intermetallic compounds presented here, their symmetries and structural characteristics.

Material	Symmetry	Unit cell parameters	Structure	Features
Ni_0.6_Pt_0.4_Bi	*P*6_3_/*mmc*	*a*=8.2745(4) Å *c*=5.4345(4) Å	“kagome‐NiAs”	2×2×1 superstructure of NiAs containing kagome mixed Ni/Pt layer and Ni interstitial layer (100 % occupancy)
Ni_0.7_Pd_0.2_Bi	*P*6_3_/*mmc*	*a*=8.1701(3) Å *c*=5.4205(3) Å	“kagome‐NiAs”	2×2×1 superstructure of NiAs containing kagome mixed Ni/Pd layer and Ni interstitial layer (86.4 % occupancy)
Mn_0.99_Pd_0.01_Bi	*P*6_3_/*mmc*	*a*=8.6010(4) Å *c*=5.7996(4) Å	“kagome‐NiAs”	2×2×1 superstructure of NiAs containing kagome mixed Mn/Pd layer and Ni interstitial layer (100 % occupancy)*
Ni_0.9_Bi	*Fmm*2	*a*=21.4180(15) Å *b*=8.1550(5) Å *c*=14.1115(8) Å	”Polar kagome‐NiAs”	Superstructure of NiAs containing kagome‐connected Ni layers, ordering of interstitial layers leading to inversion symmetry breaking. Orthorhombic strain 0.047 %
Ni_0.79_Pd_0.08_Bi	*Fmm*2	*a*=21.3794(8) Å *b*=8.1218(2) Å *c*=14.0620(4) Å	”Polar kagome‐NiAs”	Superstructure of NiAs containing kagome‐connected layers in which Ni and Pd are ordered, and ordering of interstitial layers leading to inversion symmetry breaking. Orthorhombic strain 0.019 %

* Residual amount of electron density in Mn_0.99_Pd_0.01_Bi can be modelled as partially occupied bismuth position (see Figure S8).

Two further materials are also discovered, Ni_0.9_Bi and Ni_0.79_Pd_0.08_Bi (Table 1), which both retain a kagome connectivity of transition metals in the octahedral layer but break inversion symmetry to adopt the polar space group *Fmm*2. The reflections observed in the diffraction patterns of Ni_0.9_Bi and Ni_0.79_Pd_0.08_Bi are indexed to orthorhombic unit cells, with systematic absences consistent with the *F*− − − extinction symbol. The polar space group *Fmm*2 is the only maximal subgroup of *P*6_3_/*mmc* that matches these reflection conditions. Structure solutions were unsuccessful using the non‐polar *Fmmm* symmetry as the structures of Ni_0.9_Bi and Ni_0.79_Pd_0.08_Bi cannot be modelled due to the absence of mirror symmetry orthogonal to the *c* direction. The refined Flack parameters of 0.50(3) and 0.51(2) further indicate *Fmm*2 as the correct symmetry for the structures of Ni_0.9_Bi and Ni_0.79_Pd_0.08_Bi, respectively (Table S10 and S13). The spacegroup relations are associated with the crystal chemistry; in the *P*6_3_/*mmc* space group, the structure lacks three orthogonal mirror planes. The 6_3_ screw axis defines the ABAB stacking of the hcp Bi layers. When trigonal bipyramidal interstitial sites are occupied evenly, the 6_3_ screw is retained; while there is now an in‐plane polarisation due to the interstitials in each layer (Figure S6), the 6_3_ axis relates the interstitials from one layer to the next and rotates the polarisation, leading to a net zero polarisation when the whole unit cell is considered. When the distribution of interstitials is uneven in the layers, the 6_3_ screw is now broken as the layers have different compositions. Thus the polarisation is no longer cancelled, and a net polar axis is created in the plane of the layers. It is hypothetically possible then to generate this kind of polarisation in any material that has an ABAB hcp stacking of atoms that are large enough to host trigonal bipyramidal interstitials.

**Figure 3:**
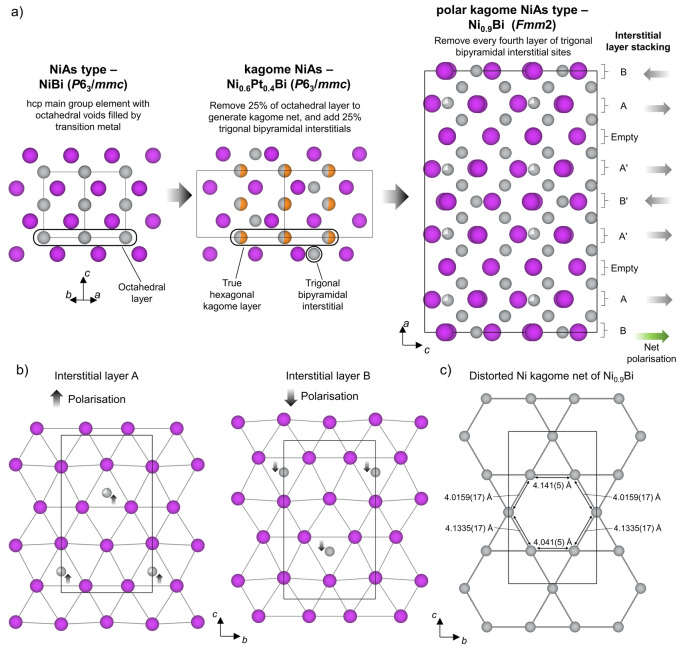
a) Structural relationship between NiAs with *P*6_3_/*mmc* symmetry (left), kagome NiAs‐type represented by Ni_0.6_Pt_0.4_Bi which has a 2×2×1 expansion of the *P*6_3_/*mmc* unit cell (middle), and polar kagome NiAs‐type represented by Ni_0.9_Bi which has *Fmm*2 symmetry (right). The structure of the polar kagome NiAs‐type material, Ni_0.9_Bi, is obtained when every fourth trigonal bipyramidal interstitial layer is empty and interstitial Ni is arranged into partially occupied layers (Layer A) and fully occupied layers (Layer B). The combination of two A and one B layers in an A‐B‐A‐empty‐A′‐B′‐A′‐empty stacking sequence along the *a* direction leads to a net polarization along the *c* axis, where the A′‐B′‐A′ set of interstitial layers is translated by half a unit cell length along the *c* axis relative to the A‐B‐A set. b) Orthogonal view of the two distinct pseudo‐hcp Bi layers (A and B) with occupied trigonal interstitial sites, each with a net polarization generated by the Ni site occupied away from the centre of the Bi rhombus. c) Pseudo‐hexagonal Ni2 layer kagome net from Ni_0.9_Bi with in‐plane distances labelled. The unit cell is shown by the black outline. The ordered Ni vacancies in the octahedral layer (dashed circles) generate the kagome net. Atom colours: Ni—grey, Pt—orange, Bi—purple.

Due to the polar distortion and subsequent orthorhombic symmetry, these materials have distorted kagome nets. These structures will be referred to as “polar kagome NiAs” for brevity. The stacking of two types of interstitial site layer in an A‐B‐A‐empty‐A′‐B′‐A′‐empty sequence (Figure 3) leads to the four‐fold expansion of the unit cell along the stacking axis (Figure S7). The A′‐B′‐A′ set of interstitial layers is translated by half a unit cell length along the *c* axis relative to the A−B−A set. The sites within the A layers are partially occupied by Ni, whereas the sites in the B layers are fully occupied. In Ni_0.9_Bi, the occupancy of the sites within the A layers is 0.698(14); the chemical formula would be Ni_0.9375_Bi if these sites were fully occupied. The occupancy of the sites within the A layers in Ni_0.79_Pd_0.08_Bi is 0.802(13). The polar symmetry in both materials is imposed by the unbalanced 2 : 1 ratio of A : B layers in the A‐B‐A‐empty‐A′‐B′‐A′‐empty stacking sequence where the A and B layers have opposing polarization (Figure 3b), generating the net polarization along *c*, despite the partial occupancy of sites within the A layers.

This type of polarization from layer ordering is distinct from that derived by displacements, and distinguishes this polar kagome‐NiAs structure from displacement‐driven polar metals such as LiOsO_3_ and the LiGaGe family of materials.[^27^, ^28^] While the polar kagome‐NiAs structure is not defined by van der Waals bonded layers, the polar axis is more akin to that in WTe_2_ in that it originates from the ordered stacking of layers,^[29]^ in this case how the interstitial transition metal layers are stacked within the structure. It is important to note that this polarity can be controlled via Pd content in the Ni−Bi system, as well as for the magnetic Mn−Bi system, as Mn_1.05_Bi adopts a polar ordering of interstitial Mn defects,^[9]^ whereas Mn_0.99_Pd_0.01_Bi adopts a non‐polar one.

**Figure 4:**
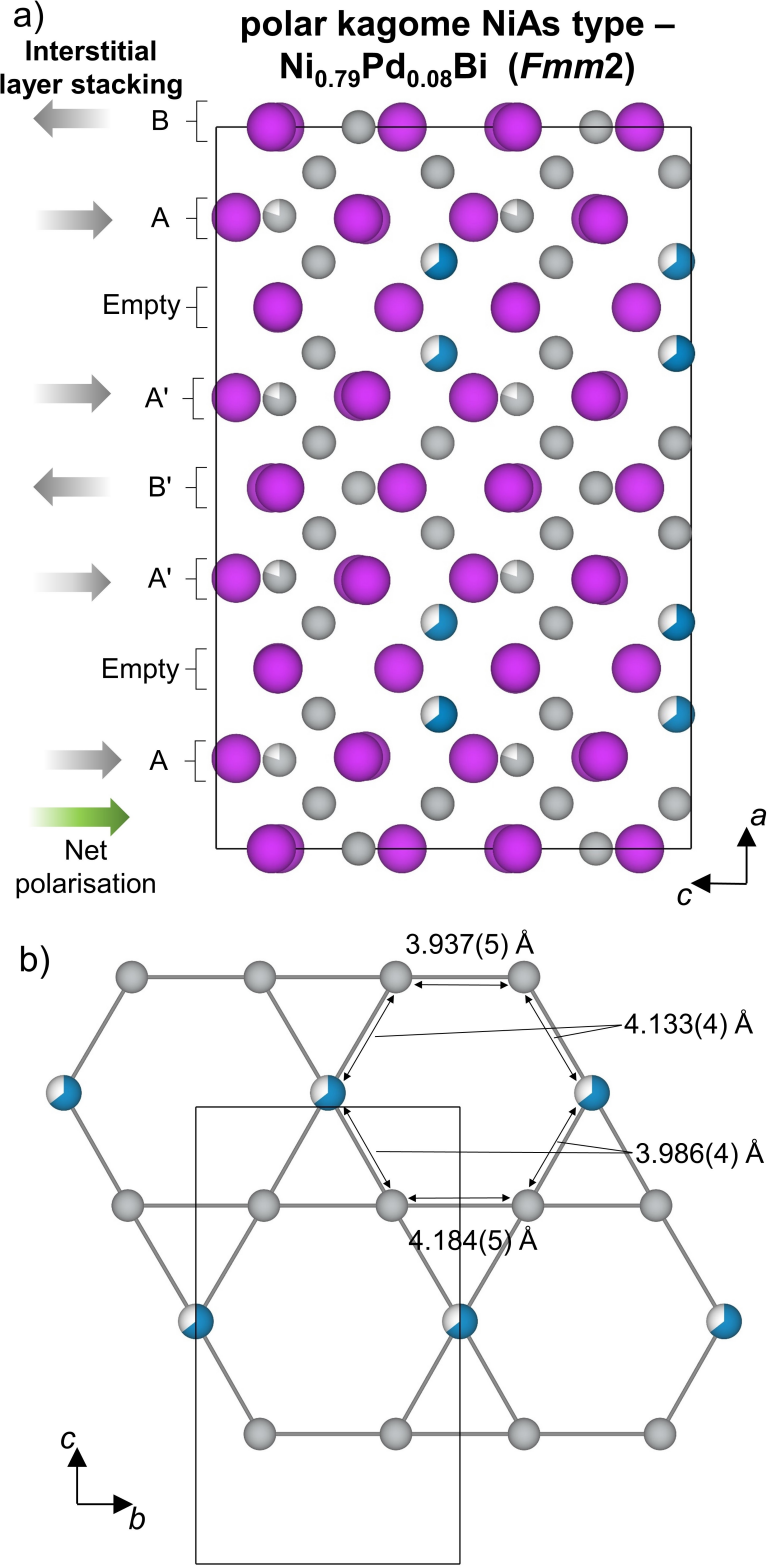
a) Crystal structure of polar kagome‐NiAs Ni_0.79_Pd_0.08_Bi with *Fmm*2 symmetry and ordering of kagome and interstitial layers along the stacking axis (*a* direction). The occupied interstitial Ni sites are arranged into partially occupied layers (Layer A) and fully occupied layers (Layer B); the absence of interstitials in half of what were originally B layers leads to a net polarization along the *c* axis. b) pseudo‐hexagonal Ni2 kagome net from Ni_0.79_Pd_0.08_Bi with fully occupied Ni and partially occupied Pd (Pd occupancy: 0.645(7)) sites occupied within the layer. The net is orthorhombically distorted and in‐plane distances are labelled. The unit cell is shown by the black outline. Atom colours: Ni—grey, Pd—blue, Bi—purple.

There are two distinct Ni1 and Ni2 kagome layers produced by the octahedral transition metal sites in both Ni_0.9_Bi or Ni_0.79_Pd_0.08_Bi (Figure 3a) which stack in a Ni1‐Ni2‐Ni2′‐Ni1′‐Ni1′‐Ni2′‐Ni2‐Ni1 pattern along the *a* axis. Both sets of layers are pseudo‐hexagonal kagome layers, with unequal nearest‐neighbour distances (Figure 3c), which are orthorhombically distorted away from the true hexagonal kagome layers observed in Ni_0.7_Pd_0.2_Bi, Ni_0.6_Pt_0.4_Bi, and Mn_0.99_Pd_0.01_Bi. There are two separate sites within each layer, which differ in their proximity to nearby occupied interstitial sites (Figure S9). Each of the two sites in both of the distorted kagome layers of Ni_0.9_Bi are fully occupied by Ni. The structure of Ni_0.79_Pd_0.08_Bi adopts the same kagome‐ and interstitial‐layer stacking as Ni_0.9_Bi (Figure 4a), and has the same Ni1 layers as Ni_0.9_Bi. However, the inclusion of Pd in Ni_0.79_Pd_0.08_Bi leads to an ordered distribution of Pd and Ni across the two separate sites within the Ni2 layer (Figure 4b), with a fully occupied Ni site, and a Pd site with 0.645(7) occupancy. This is distinct from the fully occupied Ni sites within the layers of Ni_0.9_Bi, and arises due to Pd being larger than Ni, leading to preferential occupancy of the site with greater separation from nearby occupied interstitial sites. Despite the smaller orthorhombic strain, the standard deviation of the bond lengths in Ni_0.79_Pd_0.08_Bi of 0.10 Å is greater than the 0.07 Å in Ni_0.9_Bi.

The structure of Ni_0.9_Bi is different from the previously reported Ni_1+d_Bi (space group *C*2/*m*) in both Ni content and symmetry,^[11]^ which reflects the reduced Ni content. The difference in Ni content is connected to the synthetic route with excess Bi in the Bi flux reaction, which would promote formation of a Ni‐deficient (Bi‐rich) phase. This suggests that Ni_x_Bi exists in a homogeneity range where x can be greater than, equal to, or less than one. The reduced Ni content in Ni_0.9_Bi compared to Ni_1+d_Bi leads to greater levels of vacancies in the octahedral layers, meaning all octahedral layers in Ni_0.9_Bi are kagome layers, as in the kagome‐NiAs structure, whereas in Ni_1+d_Bi, only 3/4 of the layers are kagome nets and the 25 %–100 %‐100 %–25 % occupancy pattern of interstitial layers results in no net polarisation. The incommensurate structure of Lidin et al. combines the non‐polar C2/m structure in intergrowth with *Fmm*2 motifs of the type reported here.^[30]^ Therefore modifying the Ni content in this system changes both the underlying symmetries as well as the structural motifs that are present.

**Figure 5:**
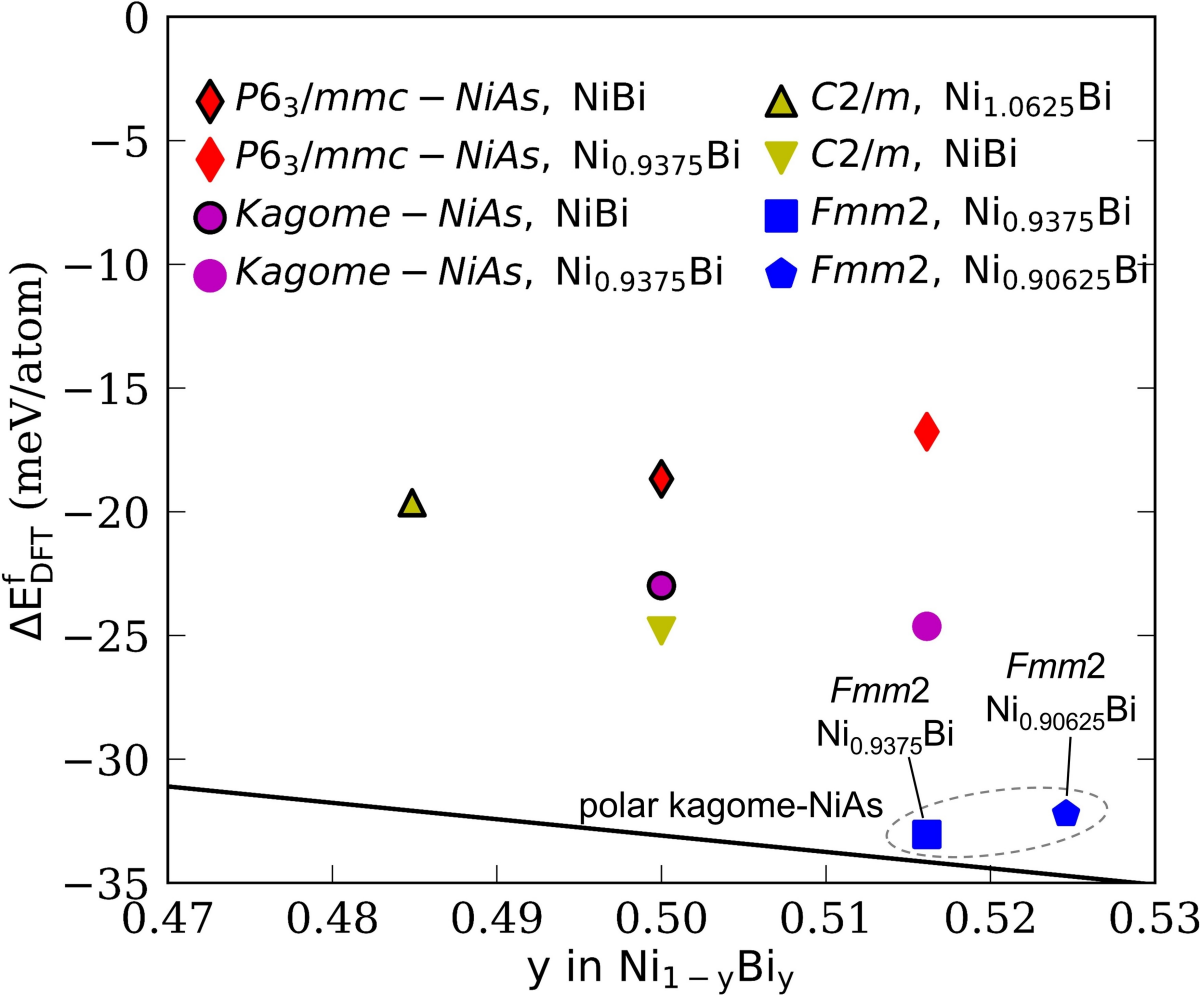
Formation energies of the Ni−Bi binary system. The black line indicates the convex hull representing phases that are thermodynamically stable at 0 K. The polar kagome‐NiAs structure in *Fmm*2 symmetry is computed to be 1 meV/atom above the convex hull at the composition Ni_0.9375_Bi. The full convex hull is provided in Figure S11 and Table S17 in the Supporting Information.

To further understand the energy landscape within the Ni−Bi system, calculations of the formation energies of competing Ni_1−x_Bi phases were performed using density functional theory (see Experimental and Supporting Information Figure S10 and Table S17). The energy of Ni_0.9375_Bi in the polar kagome‐NiAs structure is 1 meV/atom above the convex hull in good agreement with the experimental observation of Ni_0.9_Bi in this polar kagome‐NiAs structure type. The reported *C*2/*m*‐derived (ICSD‐410875) and *P*6_3_/*mmc* NiAs (ICSD‐391336) structures of stoichiometric NiBi are above the hull by 8.3 meV/atom and 14.4 meV/atom, respectively (Figure 5).[^11^, ^31^, ^32^] The kagome‐NiAs structure is 8.3 meV/atom less stable than the polar kagome‐NiAs structure of Ni_0.9375_Bi, an energy difference accessible via entropy stabilization. Considering the experimental site disorder caused by the incorporation of Pd or Pt in the kagome‐NiAs structure in Ni_0.7_Pd_0.2_Bi and Ni_0.6_Pt_0.4_Bi, the entropic contributions at 773 K are around 17 meV/atom for both compounds (see Table S18).

Vibrational and electronic properties for Ni_0.9_Bi and Ni_0.7_Pd_0.2_Bi were extracted from the heat capacity. The linear electronic components of the heat capacity for Ni_0.9_Bi and Ni_0.7_Pd_0.2_Bi are within 10 % of each other (Table 2), indicating that there is not a significant change to the density of states with Pd doping or from the change in symmetry. For the vibrational properties, two Debye temperatures are required to model the data, and likely arise from splitting of the phonon density of states into low and high frequency regions due to mass contrast between Ni and Bi^[33]^ the lower Debye temperature likely represents the vibrations of Bi, and the higher Debye temperature the vibrations of Ni/Pd.

**Table 2:** Parameters extracted from heat capacity measurements for Ni_0.9_Bi and Ni_0.7_Pd_0.2_Bi. For each material, there are two Debye temperatures (θ_D1_ and θ_D2_), one Einstein temperature (θ_E_), and the linear electronic contribution (γ). Heat capacity fits are shown in Figure S14.

Material	θ_D1_/K	θ_D2_/K	θ_E_/K	γ/mJ mol^−1^ K^−2^
Ni_0.9_Bi	140(5)	305(5)	70(5)	1.85(2)
Ni_0.7_Pd_0.2_Bi	130(5)	280(5)	65(5)	1.93(2)

The necessity of including a localized Einstein component is shown in a C_P_/T^3^ vs T plot in Figure S14. The excess heat capacity in the peak‐like feature demonstrates that Debye terms alone cannot account for the observed temperature dependence. The source of these localized phonon modes is not clear; however, localized phonon modes, or flat phonon bands, have been observed in kagome net materials such as CsV_3_Sb_5_ and CoSn, and could originate from geometric frustration of vibrational modes.[^34^, ^35^, ^36^] As seen in Table 2, the vibrational properties of both materials are similar, with slightly lower Debye and Einstein temperatures for Ni_0.7_Pd_0.2_Bi, expected due to the substitution of Ni with the heavier Pd.

From the heat capacity of the electronic term, both Ni_0.9_Bi and Ni_0.7_Pd_0.2_Bi are expected to behave as normal metals. Indeed, Ni_0.7_Pd_0.2_Bi shows the standard temperature dependence of resistivity of a metal, and can be fit well by a modified Bloch‐Gruneisen function (see supplementary material) with *n*=5, typical of a simple metal with low temperature Fermi liquid behavior (with the inclusion of an Einstein term *T_E_* =57(3) K and a Bloch temperature, or the temperature characteristic of the electron‐phonon scattering, *T_B_* =161(2) K). In contrast, although the resistivity of Ni_0.9_Bi is metallic at all measured temperatures, the resistivity below 150 K decreases without approaching a low‐temperature plateau, inconsistent with conventional metallic behaviour. It thus could not be fit with a standard or modified Bloch‐Gruneisen function or a *T* ^2^ law for typical Fermi liquids. This is indicative of unconventional electronic behavior rather than a structural phase transition;^[37]^ and indeed no transition is observed in the heat capacity (Figure S14).

**Figure 6:**
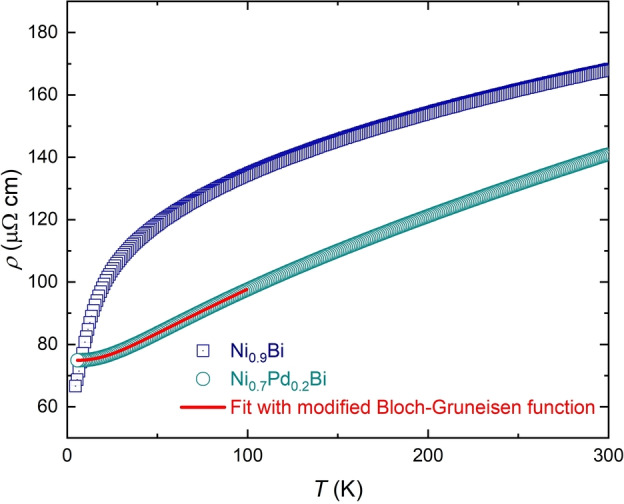
Resistivity of Ni_0.9_Bi (blue squares) and Ni_0.7_Pd_0.2_Bi (cyan circles). For Ni_0.7_Pd_0.2_Bi, the modified Bloch‐Gruneisen fit to the resistivity from 5 K to 100 K is shown in the red line. For both materials, the current was applied along the *c*‐axis.

Both were determined to be non‐magnetic metals by temperature dependent resistivity and magnetisation measurements performed on single crystals. The resistivity measurements are shown in Figure 6; the magnetisation measurements for both materials show diamagnetism at room temperature with a small Curie tail developing at low temperatures (Figure S13). Due to sample geometry (needle‐like for Ni_0.7_Pd_0.2_Bi, and plate‐like for both Ni_0.9_Bi), the resistivity in Ni_0.9_Bi was measured in the *bc*‐plane (perpendicular to the stacking axis) and in Ni_0.7_Pd_0.2_Bi was measured along the *c*‐axis (along the stacking axis). The room temperature resistivities of both materials are of the same order of magnitude.

This difference in resistivity behavior with temperature, and the unconventional nature of Ni_0.9_Bi, cannot be explained through the vibrational properties, or through large changes to the density of states, given the similarity of the properties as extracted from heat capacity. Therefore, it is attributable to the structure, and likely the polar axis present in Ni_0.9_Bi. This polar axis, in the presence of spin‐orbit coupling, spin‐splits the electronic bands which can lead to potential Weyl nodes,^[38]^ enhancement of Berry curvature,[^24^, ^39^, ^40^] and small pockets on the Fermi surface.[^41^, ^42^, ^43^] These effects tend not to lead to large‐scale changes in the density of states, but can drastically affect the electron scattering. Therefore, it is likely that the chemically controllable polar axis in these kagome‐NiAs materials can act as an on/off switch for unconventional metallic behaviour.

## Conclusion

Synthesis in bismuth fluxes affords a family of superstructures of the well‐known NiAs structure type in which coupled formation of vacancies and interstitials generates kagome transition metal layers, along with compositionally tuneable inversion symmetry breaking. This is consistent with convex hull calculations that show the bismuthide phase diagrams to be energetically flat near the 1 : 1 ratio. Further defect ordering within this family introduces a polar axis into the kagome‐connected layers. The unconventional electronic transport in polar Ni_0.9_Bi contrasts with the conventional metallic behavior of the non‐polar members of this materials family, and points to potentially extensive compositional control of structure and properties in a chemistry that affords the heavily studied kagome net.

## Supporting Information

Supplementary files include crystallographic information files for each of the five structures. The Supporting Information file contains details of the crystal structures of the five new materials reported here, energy dispersive X‐ray spectroscopy data, DFT calculations, magnetization data, electronic transport data, and heat capacity data measured from the compounds reported here.

Deposition Numbers https://www.ccdc.cam.ac.uk/services/structures?id=doi:10.1002/anie.202403670 2298182 (for Ni_0.6_Pt_0.4_Bi), 2298183 (for Ni_0.7_Pd_0.2_Bi), 2298184 (for Mn_0.99_Pd_0.01_Bi), 2298185 (for Ni_0.9_Bi), 2298186 (for Ni_0.79_Pd_0.08_Bi) contain the supplementary crystallographic data for this paper. These data are provided free of charge by the joint Cambridge Crystallographic Data Centre and Fachinformationszentrum Karlsruhe http://www.ccdc.cam.ac.uk/structures Access Structures service.

Underlying data collected as part of this work is available at the University of Liverpool data repository at https://datacat.liverpool.ac.uk/id/eprint/2470.

## Conflict of interests

The authors declare no conflict of interest.

1

## Supporting information

As a service to our authors and readers, this journal provides supporting information supplied by the authors. Such materials are peer reviewed and may be re‐organized for online delivery, but are not copy‐edited or typeset. Technical support issues arising from supporting information (other than missing files) should be addressed to the authors.

Supporting Information

Supporting Information

Supporting Information

Supporting Information

Supporting Information

Supporting Information

## Data Availability

The data that support the findings of this study are openly available in University of Liverpool Data Repository at https://datacat.liverpool.ac.uk/cgi/users/home?screen=EPrint::View&eprintid=2470, reference number 2470.
