# Supplementary material for: Control of Polarity in Kagome‐NiAs Bismuthides
Source: Angew Chem Int Ed Engl. 2024 Apr 3;63(23):e202403670. doi: 10.1002/anie.202403670 (PMC11497289; doi:10.1002/anie.202403670)
Supplement: Supplementary file 1 — Supporting Information [file ANIE-63-e202403670-s006.pdf]

Angewandte Chemie
Eine Zeitschrift der Gesellschaft Deutscher Chemiker

Supporting Information

Control of Polarity in Kagome-NiAs Bismuthides

*Q. D. Gibson, D. Wen, H. Lin, M. Zanella, L. M. Daniels, C. M. Robertson, J. B. Claridge, J. Alaria, M. S. Dyer, M. J. Rosseinsky**

Supporting Information
©Wiley-VCH 2021
69451 Weinheim, Germany

Control of Polarity in Kagome-NiAs Bismuthides

Quinn D. Gibson, Dongsheng Wen, Hai Lin, Marco Zanella, Luke M. Daniels, Craig M. Robertson, John B. Claridge, Jonathan Alaria, Matthew S. Dyer, Matthew J. Rosseinsky*

Table of Contents

Item	Pages
Experimental Procedures	2
Density functional theory calculations	3
Crystal structure of kagome-NiAs type $Ni_{0.6}Pt_{0.4}Bi$	4-5
Crystal structure of kagome-NiAs type $Ni_{0.7}Pd_{0.2}Bi$	6-7
Crystal structure of kagome-NiAs type $Mn_{0.99}Pd_{0.01}Bi$	8-9
Crystal structure of polar kagome-NiAs type $Ni_{0.9}Bi$	10-11
Crystal structure of polar kagome-NiAs type $Ni_{0.79}Pd_{0.08}Bi$	12-13
Energy dispersive X-ray spectroscopy	14-16
Structural discussion	17-19
Energy calculations	20-23
Magnetisation measurements	24
Electronic transport measurements	24
Heat capacity measurements	25
References	26

Experimental Procedures

For all syntheses, the metals (Ni powder, Mn chunk, Pd chunk, Pt wire and Bi pieces) were sealed in an evacuated quartz tube. This was heated to 1000 °C for 12 h before being cooled at a rate of 0.1 °C/min to 500 °C (for Ni containing compounds) or 430 °C (for Mn containing compounds), at which point the tube was centrifuged to remove excess Bi flux.
Energy dispersive X-ray analysis (EDX) was performed to confirm the compositions obtained from single crystal X-ray refinement.

Single crystals of $Ni_{0.6}Pt_{0.4}Bi$, $Ni_{0.79}Pd_{0.2}Bi$, $Mn_{0.99}Pd_{0.01}Bi$, $Ni_{0.9}Bi$, and $Ni_{0.79}Pd_{0.08}Bi$ were selected for single crystal X-ray diffraction. A suitable crystal was broken from a larger crystal using a scalpel. The crystals were mounted on MiTeGen tips using Parabar oil and placed on a Rigaku 007HF Mo rotating anode single crystal diffractometer. The crystal was kept at 100.00(10) K during data collection. Using Olex2,[1] the structure was solved with the ShelXT[2] structure solution program using Intrinsic Phasing and refined with the ShelXL[3] refinement package using Least Squares minimisation. Owing to the strong X-ray absorption of Bi, large residual electron density values were observed within its proximity. Due to the small crystal dimensions of each sample measured (average lengths < 20 μm), face indexing was not possible and only a standard multi-scan absorption correction was applied.

$Ni_{0.9}Bi$ was synthesized by a Bi self-flux reaction, with a Ni:Bi ratio of 1:4. The EDX composition was determined to be $Ni_{0.87(2)}Bi$, which is within two standard deviation error of the single crystal composition of $Ni_{0.899(4)}Bi$
$Ni_{0.79}Pd_{0.08}Bi$ was synthesized by a Bi self-flux reaction with a Ni:Pd:Bi ratio of 17:3:80. The EDX composition was determined to be $Ni_{0.80(2)}$ $Pd_{0.04(2)}Bi$. Both $Ni_{0.9}Bi$ and $Ni_{0.79}Pd_{0.08}Bi$ form as large (facets > 1mm) chunky crystals with pseudo-hexagonal facets.
$Ni_{0,7}Pd_{0.2}Bi$ was synthesized with a Ni:Pd:Bi ratio of 1:1:8. $Ni_{0,7}Pd_{0.2}Bi$ grows as needle-like crystals with dimensions of about 0.2mm thickness and 1-2mm length. The EDX composition was determined to be $Ni_{0.73(2)}Pd_{0.19(1)}Bi$, which is within two standard deviations of the single crystal composition of $Ni_{0.7}Pd_{0.2}Bi$. This will be written as $Ni_{0.7}Pd_{0.2}Bi$ for convenience.
$Ni_{0.6}Pt_{0.4}Bi$ was synthesized by a Bi flux reaction with a. Ni:Pt:Bi ratio of 1:1:8. $Ni_{0.6}Pt_{0.4}Bi$ crystallises as flake-like crystals. From the single crystal refinement, the formula was refined to $Ni_{2.41(3)}Pt_{1.59(3)}Bi_4$, with no vacancies and which is within error of the composition $Ni_{0.6}Pt_{0.4}Bi$, which is written for simplicity, and is consistent with the EDX results.
$Mn_{0.99}Pd_{0.01}Bi$ was synthesized by a Bi self-flux reaction, with a Mn:Pd:Bi ratio of 17:3:80. $Mn_{0.99}Pd_{0.01}Bi$ grows as very small needles of less than 0.1 mm in length, coexisting with large crystals (with facets >0.1 mm) of a competing Mn-Pd-Bi phase. $Mn_{0.99}Pd_{0.01}Bi$ can be separated mechanically from these larger, chunkier crystals. From EDX, the composition of Pd doped MnBi is $Mn_{0.986(9)}Pd_{0.014(9)}$, consistent with the single crystal compositions of $Mn_{0.99(1)}Pd_{0.01(1)}Bi$ and $Mn_{1.05(3)}Pd_{0.038(15)}Bi$ measured on two different single crystal samples (Supplementary Information). As such, we will use the composition of $Mn_{0.99}Pd_{0.01}Bi$, with the understanding that there is some variation of Pd content from crystal to crystal, and that the Mn content depends on whether the excess electron density is modelled as partially occupied Mn or Bi.
The electrical transport and heat capacity measurements were performed on Quantum Design Physical Properties Measurement System (QD-PPMS-DynaCool). The resistivities were all measured with the 4-probe method, applying a DC current of 2 mA. Heat capacity was measured using the relaxation method with the crystal mounted on the sample puck with N-grease. The contribution measured from the puck and the grease was subtracted from the results to obtain the heat capacity of the material.

Density functional theory calculations

The stabilities of the different structure types in the Ni-Bi system were studied by calculating the formation energy using density functional theory:

$$\Delta E^{f}_{DFT} = E_{DFT}\left(Ni_{1-y}B_{y}\right) - (1-y)E_{DFT}(Ni) - yE_{DFT}(Bi)$$

in which $E_{DFT}\left(Ni_{1-y}Bi_{y}\right)$ is the total energy per atom of the structure of interest for the composition of $Ni_{1-y}Bi_{y}$ (Figure S10), and $E_{DFT}(Ni)$ and $E_{DFT}(Bi)$ are the ground-state energies per atom of $Fm\overline{3}m$ Ni, and $Pm\overline{3}m$ Bi respectively. All the reported structures of the Ni-Bi system on ICSD and Materials Project were calculated using the Vienna Ab-initio Simulation Package (VASP) with the PBE exchange-correlation functional.[4-6] Where structures were initially disordered, or where vacancies have been incorporated to generate structures at different compositions, ordered cells were created using CASMcode[7] to enumerate symmetrically distinct Ni-vacancy structures with the cell compositions close to NiBi and the lowest energy ordering used for analysis. This process is discussed in more depth in the supplementary information and all structures used are reported in Table S17 and Figure S11. The Ni 3*p*, and Bi 6*s* electrons were considered as valence states. The projector augmented wave method was used with a global energy cutoff of 520 eV.[4] The automatic *k*-mesh scheme was used with R_k = 40 Å. Colinear spin polarization was allowed and the initial magnetic moments for Ni and Bi were set to 1 and 0 μ_B, respectively. Ionic and volume optimizations were carried out until the magnitude of forces on all atoms fell within 0.001 eV/Å, and the electronic self-consistent loops were converged within 10^{-10} eV.

Results and Discussion

Crystal structure of $Ni_{0.6}Pt_{0.4}Bi$

Table S1. Crystal data and structure refinement for $Ni_{0.6}Pt_{0.4}Bi$.

Identification code	$Ni_{0.6}Pt_{0.4}Bi$
Empirical formula	$Bi_2Ni_{1.21}Pt_{0.8}$
Formula weight	643.80
Temperature/K	100.00(10)
Crystal system	hexagonal
Space group	$P6_3/mmc$
a/Å	8.2745(4)
b/Å	8.2745(4)
c/Å	5.4345(4)
α/°	90
β/°	90
γ/°	120
Volume/Å^3	322.24(4)
Z	4
ρ_{calc}g/cm^3	13.271
μ/mm^{-1}	149.919
F(000)	1047.0
Crystal size/mm^3	0.022 × 0.02 × 0.02
Radiation	Mo Kα (λ = 0.71073)
2Θ range for data collection/°	5.686 to 61.272
Index ranges	$-10 \le h \le 10$, $-11 \le k \le 10$, $-6 \le l \le 7$
Reflections collected	3346
Independent reflections	198 [R_{int} = 0.0488, R_{sigma} = 0.0218]
Data/restraints/parameters	198/0/15
Goodness-of-fit on F^2	1.116
Final R indexes [I>=2σ (I)]	R_1 = 0.0232, wR_2 = 0.0536
Final R indexes [all data]	R_1 = 0.0249, wR_2 = 0.0540
Largest diff. peak/hole / e Å^{-3}	2.76/-4.69

Table S2. Fractional Atomic Coordinates (×10^4) and Equivalent Isotropic Displacement Parameters (Å^2×10^3) for $Ni_{0.6}Pt_{0.4}Bi$. U_{eq} is defined as 1/3 of the trace of the orthogonalised U_{IJ} tensor.

**Atom**	***x***	***y***	***z***	**U(eq)**	**Occ.**
Bi01	1497.8(6)	2995.6(11)	7500	5.2(3)	1
Bi02	3333.33	6666.67	2500	10.0(4)	1
Pt03	5000	5000	10000	5.1(5)	0.530(9)
Ni04	3333.33	6666.67	7500	9.9(10)	1
NiA	5000	5000	10000	5.1(5)	0.470(9)

Table S3. Anisotropic Displacement Parameters (Å^2×10^3) for $Ni_{0.6}Pt_{0.4}Bi$. The Anisotropic displacement factor exponent takes the form: $-2\pi^2[h^2a^{*2}U_{11}+2hka^*b^*U_{12}+\dots]$.

**Atom**	**U_{11}**	**U_{22}**	**U_{33}**	**U_{23}**	**U_{13}**	**U_{12}**
Bi01	5.9(4)	6.4(4)	3.4(4)	0	0	3.2(2)
Bi02	10.2(5)	10.2(5)	9.5(7)	0	0	5.1(2)
Pt03	5.8(6)	5.8(6)	4.3(7)	0.1(2)	-0.1(2)	3.5(5)
Ni04	9.5(15)	9.5(15)	11(3)	0	0	4.7(7)
NiA	5.8(6)	5.8(6)	4.3(7)	0.1(2)	-0.1(2)	3.5(5)

2 Crystal structure of $Ni_{0.7}Pd_{0.2}Bi$

Table S4. Crystal data and structure refinement for $Ni_{0.7}Pd_{0.2}Bi$.

Identification code	$Ni_{0.7}Pd_{0.2}Bi$.
Empirical formula	$Bi_{1.33}Ni_{1.04}Pd_{0.24}$
Formula weight	365.88
Temperature/K	100.00(10)
Crystal system	hexagonal
Space group	$P6_3/mmc$
a/Å	8.1701(3)
b/Å	8.1701(3)
c/Å	5.4205(3)
α/°	90
β/°	90
γ/°	120
Volume/Å^3	313.35(3)
Z	6
ρ_{calc}g/cm^3	11.634
μ/mm^{-1}	123.001
F(000)	907.0
Crystal size/mm^3	0.2 × 0.2 × 0.15
Radiation	Mo Kα (λ = 0.71073)
2Θ range for data collection/°	5.758 to 61.488
Index ranges	$-10 \leq h \leq 10$, $-11 \leq k \leq 10$, $-6 \leq l \leq 7$
Reflections collected	3441
Independent reflections	205 [R_{int} = 0.0521, R_{sigma} = 0.0246]
Data/restraints/parameters	205/0/16
Goodness-of-fit on F^2	1.223
Final R indexes [I>=2σ (I)]	R_1 = 0.0180, wR_2 = 0.0373
Final R indexes [all data]	R_1 = 0.0214, wR_2 = 0.0379
Largest diff. peak/hole / e Å^{-3}	2.84/-2.37

Table S5. Fractional Atomic Coordinates ($\times10^4$) and Equivalent Isotropic Displacement Parameters ($Å^2\times10^3$) for $Ni_{0.7}Pd_{0.2}Bi$. U_{eq} is defined as 1/3 of of the trace of the orthogonalised U_{IJ} tensor.

**Atom**	***x***	***y***	***z***	**U(eq)**	**Occ.**
Bi01	3333.33	6666.67	7500	5.7(2)	1
Bi02	1506.9(3)	3013.9(7)	2500	7.85(19)	1
Ni03	0	5000	0	5.9(5)	0.757(15)
Ni04	3333.33	6666.67	2500	6.9(10)	0.864(14)
Pd01	0	5000	0	5.9(5)	0.243(15)

Table S6. Anisotropic Displacement Parameters ($Å^2\times10^3$) for $Ni_{0.7}Pd_{0.2}Bi$. The Anisotropic displacement factor exponent takes the form: $-2\pi^2[h^2a^{*2}U_{11}+2hka^*b^*U_{12}+\ldots]$.

**Atom**	**U_{11}**	**U_{22}**	**U_{33}**	**U_{23}**	**U_{13}**	**U_{12}**
Bi01	4.4(2)	4.4(2)	8.5(4)	0	0	2.19(12)
Bi02	6.9(2)	9.9(3)	7.7(3)	0	0	4.93(13)
Ni03	4.0(7)	5.5(6)	7.7(8)	0.2(2)	0.4(4)	2.0(3)
Ni04	4.3(11)	4.3(11)	12.1(17)	0	0	2.2(6)
Pd01	4.0(7)	5.5(6)	7.7(8)	0.2(2)	0.4(4)	2.0(3)

3 Crystal structure of $Mn_{0.99}Pd_{0.01}Bi$

Table S7. Crystal data and structure refinement for $Mn_{0.99}Pd_{0.01}Bi$.

Identification code	W0261_auto
Empirical formula	$Bi_{2.052}Mn_{1.975}Pd_{0.025}$
Formula weight	540.10
Temperature/K	100.00(10)
Crystal system	hexagonal
Space group	$P6_3/mmc$
a/Å	8.6010(4)
b/Å	8.6010(4)
c/Å	5.7996(4)
α/°	90
β/°	90
γ/°	120
Volume/Å^3	371.56(4)
Z	4
ρ_{calc}g/cm^3	9.807
μ/mm^{-1}	103.661
F(000)	884
Crystal size/mm^3	0.02 × 0.02 × 0.01
Radiation	Mo Kα (λ = 0.71073)
2Θ range for data collection/°	5.47 to 60.35
Index ranges	$-8 \leq h \leq 11$, $-10 \leq k \leq 8$, $-6 \leq l \leq 7$
Reflections collected	2600
Independent reflections	215 [R_{int} = 0.0390, R_{sigma} = 0.0219]
Data/restraints/parameters	215/0/15
Goodness-of-fit on F^2	1.170
Final R indexes [I>=2σ (I)]	R_1 = 0.0233, wR_2 = 0.0506
Final R indexes [all data]	R_1 = 0.0248, wR_2 = 0.0510
Largest diff. peak/hole / e Å^{-3}	2.250/-4.190

Table S8. Fractional Atomic Coordinates (×10^4) and Equivalent Isotropic Displacement Parameters (Å^2×10^3) for $Mn_{0.99}Pd_{0.01}Bi$. U_{eq} is defined as 1/3 of the trace of the orthogonalised U_{IJ} tensor.

**Atom**	***x***	***y***	***z***	**U(eq)**	**Occ.**
Bi01	7069.4(10)	8534.7(5)	7500	6.3(3)	1
Bi02	3333.33	6666.67	2500	8.3(3)	1
Mn03	5000	5000	5000	73(10)	0.983(17)
Mn04	3333.33	6666.67	7500	14.7(12)	1
Pd01	5000	5000	5000	73(10)	0.017(17)
Bi1	2260(20)	4520(50)	2500	29(11)	0.035(5)

Table S9. Anisotropic Displacement Parameters (Å^2×10^3) for Mn09Pt01Bi. The Anisotropic displacement factor exponent takes the form: $-2\pi^2[h^2a^{*2}U_{11}+2hka^*b^*U_{12}+\ldots]$.

**Atom**	**U_{11}**	**U_{22}**	**U_{33}**	**U_{23}**	**U_{13}**	**U_{12}**
Bi01	5.0(4)	6.9(3)	6.3(4)	0	0	2.50(19)
Bi02	8.9(4)	8.9(4)	7.5(6)	0	0	4.4(2)
Mn03	8.2(12)	8.2(12)	4.5(16)	1.2(5)	-1.2(5)	3.3(11)
Pd01	8.2(12)	8.2(12)	4.5(16)	1.2(5)	-1.2(5)	3.3 (11)

4 Crystal structure of $Ni_{0.9}Bi$

Table S10. Crystal data and structure refinement for $Ni_{0.9}Bi$.

Identification code	$Ni_{0.9}Bi$.
Empirical formula	$Bi_{12.8}Ni_{11.5}$
Formula weight	365.88
Temperature/K	287.00(11)
Crystal system	orthorhombic
Space group	Fmm2
a/Å	21,4180(15)
b/Å	8.1550(5)
c/Å	14.1115(8)
α/°	90
β/°	90
γ/°	90
Volume/Å^3	2464.8(3)
Z	1
ρ_{calc}g/cm^3	11.288
μ/mm^{-1}	124.406
F(000)	6924.4
Crystal size/mm^3	0.05 × 0.02 × 0.01
Radiation	Mo Kα (λ = 0.71073)
2Θ range for data collection/°	3.804 to 61.982
Index ranges	$-30 \leq h \leq 29$, $-11 \leq k \leq 11$, $-19 \leq l \leq 19$
Reflections collected	15387
Independent reflections	1934 [R_{int} = 0.0555, R_{sigma} = 0.0274]
Data/restraints/parameters	1866/0/82
Goodness-of-fit on F^2	1.206
Final R indexes [I>=2σ (I)]	R_1 = 0.0256, wR_2 = 0.0642
Final R indexes [all data]	R_1 = 0.0269, wR_2 = 0.0648
Largest diff. peak/hole / e Å^{-3}	3.9/-3.2
Flack parameter	0.50(3)

Table S11. Fractional Atomic Coordinates (×10[4]) and Equivalent Isotropic Displacement Parameters (Å[2]×10[3]) for $Ni_{0.9}Bi$. U_{eq} is defined as 1/3 of of the trace of the orthogonalized U_{IJ} tensor.

Atom	*x*	*y*	*z*	U(eq)	Occ.
Bi01	0.62709(4)	0.500000	0.29954(9)	4.2(3)	1
Bi02	0.500000	0.77596(13)	0.39157(9)	5.6(3	1
Bi03	0.500000	0.500000	0.11558(12)	6.3(4)	1
Bi04	0.750000	0.250000	0.88484(10)	6.1(3)	1
Bi05	0.75017(5)	0.500000	0.63037(9)	6.0(3)	1
Bi06	0.62499(2)	0.23125(12)	0.54288(7)	10.7(3)	1
Bi07	0.62738(4)	0.500000	0.81295(12)	11.1(4)	1
Bi08	0.500000	0.500000	0.63378(13)	4.3(4)	1
Ni09	0.500000	0.500000	0.3000(4)	5.5(9)	1
Ni0A	0.56202(16)	0.500000	0.4660(5)	11.9(5)	1
Ni0B	0.56236(12)	0.2418(6)	0.7167(4)	7.4(4)	1
Ni0C	0.68603(11)	0.500000	0.4692(3)	0.2(5)	1
Ni0D	0.68752(13)	0.2539(4)	0.7155(5)	9.2(4)	1
Ni1	0.62262(16)	0.500000	0.6339(4)	5.9(14)	0.698(14)

Table S12. Anisotropic Displacement Parameters (Å[2]×10[3]) for $Ni_{0.9}Bi$. The Anisotropic displacement factor exponent takes the form: $-2\pi^2[h^2a^{*2}U_{11}+2hka^*b^*U_{12}+\ldots]$.

Atom	U_{11}	U_{22}	U_{33}	U_{23}	U_{13}	U_{12}
Bi01	3.7(5)	4.8(7)	4.2(8)	0	0.6(2)	0
Bi02	5.9(4)	5.3(5)	5.6(8)	-0.1(3)	0	0
Bi03	7.7(6)	6.7(9)	4.5(10)	0	0	0
Bi04	4.9(4)	8.8(4)	4.7(7)	0	0	-2.1(2)
Bi05	5.9(4)	4.3(4)	7.8(8)	0	0.1(2)	0
Bi06	8.3(4)	14.1(5)	9.7(8)	3.5(2)	-0.2(2)	0.0(16)
Bi07	8.2(5)	7.3(7)	17.9(10)	0	0.8(3)	0
Bi08	6.4(6)	3.5(9)	2.9(9)	0	0	0
Ni09	6.2(18)	8(2)	3(2)	0	0	0
Ni0A	9.7(10)	15.7(12)	10.4(11)	0	-0.8(6)	0
Ni0B	7.7(7)	4.2(10)	10.4(8)	3.6(4)	0.5(4)	-0.5(4)
Ni1	9(2)	4(3)	5(3)	0	-0.2(9)	0

5 Crystal structure of $Ni_{0.79}Pd_{0.08}Bi$

Table S13. Crystal data and structure refinement for $Ni_{0.79}Pd_{0.08}Bi$.

Identification code	W0200_auto
Empirical formula	$Bi_{2.21}Ni_{1.74}Pd_{0.18}$
Formula weight	582.15
Temperature/K	100.01(10)
Crystal system	orthorhombic
Space group	Fmm2
a/Å	21.3794(8)
b/Å	8.1218(2)
c/Å	14.0620(4)
α/°	90
β/°	90
γ/°	90
Volume/Å^3	2441.72(13)
Z	29
ρ_{calc}g/cm^3	11.481
μ/mm^{-1}	125.159
F(000)	6960.0
Crystal size/mm^3	0.2 × 0.2 × 0.2
Radiation	Mo Kα (λ = 0.71073)
2Θ range for data collection/°	3.81 to 60.782
Index ranges	$-28 \leq h \leq 28, -10 \leq k \leq 8, -17 \leq l \leq 17$
Reflections collected	6791
Independent reflections	1596 [R_{int} = 0.0261, R_{sigma} = 0.0192]
Data/restraints/parameters	1596/13/76
Goodness-of-fit on F^2	1.147
Final R indexes [I>=2σ (I)]	R_1 = 0.0213, wR_2 = 0.0530
Final R indexes [all data]	R_1 = 0.0219, wR_2 = 0.0531
Largest diff. peak/hole / e Å^{-3}	3.74/-2.98
Flack parameter	0.51(2)

Table S14. Fractional Atomic Coordinates (×10^4) and Equivalent Isotropic Displacement Parameters (Å^2×10^3) for $Ni_{0.79}Pd_{0.08}Bi$. U_{eq} is defined as 1/3 of of the trace of the orthogonalised U_{IJ} tensor.

**Atom**	***x***	***y***	***z***	**U(eq)**	**Occ.**
Bi01	3732.3(3)	5000	7006.0(6)	0.5(3)	1
Bi02	3717.3(4)	5000	1847.2(8)	5.4(3)	1
Bi03	3749.2(2)	7709.0(10)	4579.8(4)	5.4(2)	1
Bi04	5000	2244.1(11)	6087.5(6)	1.0(2)	1
Bi05	2500	7500	1153.5(7)	2.0(2)	1
Bi06	5000	5000	8837.6(9)	2.2(3)	1
Bi07	2490.6(4)	5000	3699.4(6)	1.9(2)	1
Bi08	5000	5000	3662.2(10)	2.1(3)	1
Ni09	5000	5000	7008(3)	2.3(8)	1
Ni0A	4377.3(15)	5000	5335(3)	3.7(4)	1
Ni0B	3126.8(12)	7422(3)	2843(2)	2.0(3)	1
Ni0C	4372.5(12)	7507(6)	2833(2)	1.6(3)	1
Pd0E	3136.6(11)	5000	5308(2)	2.0(7)	0.645(7)
Ni1	3769.9(13)	5000	3670(3)	3.7(11)	0.802(13)

Table S15. Anisotropic Displacement Parameters (Å^2×10^3) for $Ni_{0.79}Pd_{0.08}Bi$. The Anisotropic displacement factor exponent takes the form: $-2\pi^2[h^2a^{*2}U_{11}+2hka^*b^*U_{12}+\ldots]$.

**Atom**	**U_{11}**	**U_{22}**	**U_{33}**	**U_{23}**	**U_{13}**	**U_{12}**
Bi02	4.1(4)	2.6(4)	9.6(5)	0	1.1(3)	0
Bi03	2.8(4)	7.3(4)	5.9(4)	3.1(3)	-0.10(18)	0.0(2)
Bi04	1.1(5)	1.0(5)	0.8(5)	0.1(4)	0	0
Bi05	0.6(4)	3.0(3)	2.4(4)	0	0	-0.7(3)
Bi06	1.0(5)	0.9(6)	4.9(8)	0	0	0
Ni0A	2.6(10)	4.6(10)	4.1(9)	0	-0.2(8)	0
Ni0B	1.1(7)	1.9(7)	3.1(7)	0.5(8)	-0.4(5)	0.5(9)
Pd0E	3.2(11)	1.6(10)	1.1(10)	0	0.9(7)	0
Ni1	3.8(17)	0.9(18)	6.3(17)	0	-0.5(10)	0

6 Energy dispersive X-ray spectroscopy

$Ni_{0.9}Bi$

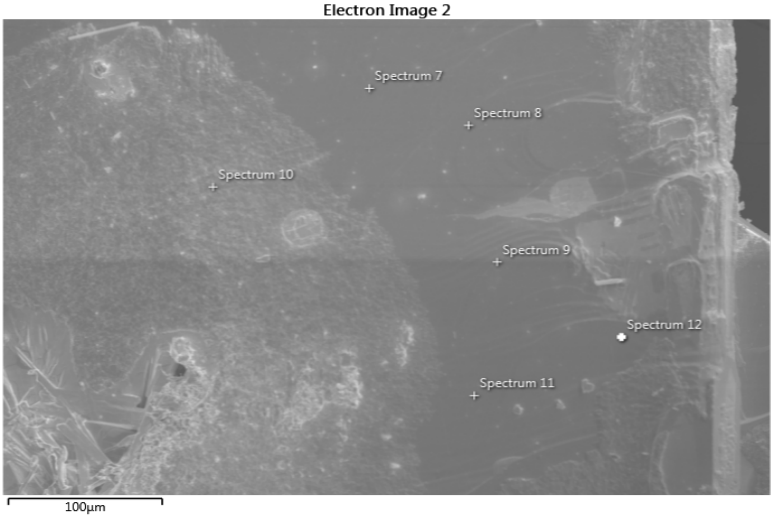

Figure S1. An SEM image of the surface of a large $Ni_{0.9}Bi$ crystal. Points at which EDX spectra were recorded are shown.

For $Ni_{0.9}Bi$, multiple EDX spectra were collected at different points on the surface of a single crystal. Spectrum 10 contains an excess of bismuth, due to a small amount of Bi flux left on the surface – this can also be seen by a slight increase in brightness in these areas. The average Ni:Bi ratio from the remaining spectra is 0.87(2):1 which corresponds to a composition of $Ni_{0.87(2)}Bi$, which is within two standard deviations of the single crystal composition.

$Ni_{0.79}Pd_{0.08}Bi$

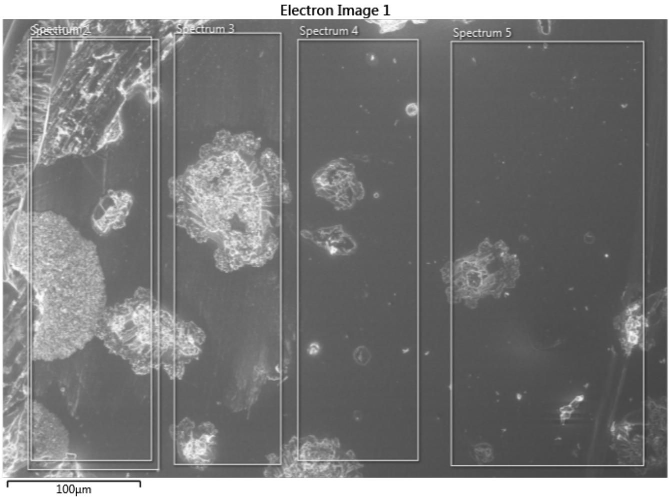

Figure S2. An SEM image of the surface of a large $Ni_{0.79}Pd_{0.08}Bi$ crystal. Points at which EDX spectra were recorded are shown.

Spectra 1 and 2 have excess Bi due to incomplete removal of the Bi flux. From the average of the spectra 3 and 4 on the crystal surface, the average Ni:Pd:Bi ratio is determined to be 0.80(1):0.04(2):1, giving a composition of $Ni_{0.80(1)}Pd_{0.04(2)}Bi$. The Pd content is lower than that obtained from single crystal data, but is within two standard deviations. It must be noted that the Pd signal is close to the sensitivity floor EDX in this case, and as such quantitative values are expected to have high error.

$Ni_{0.7}Pd_{0.2}Bi$

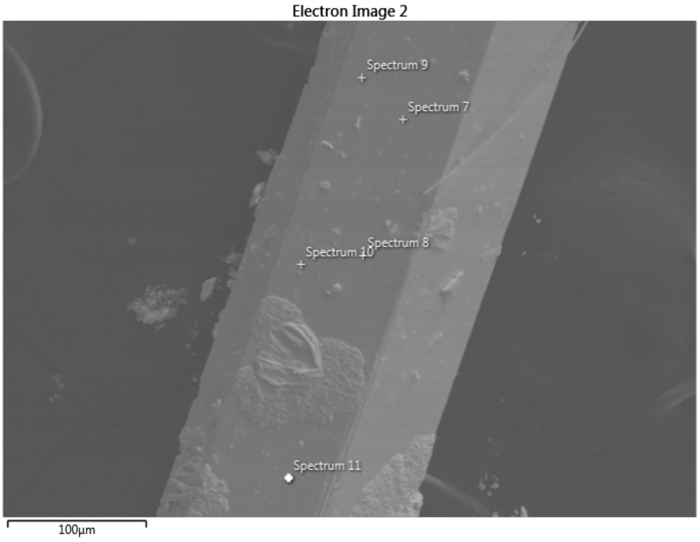

Figure S3. An SEM image of the surface of a large rod-like $Ni_{0.7}Pd_{0.2}Bi$ crystal. Points at which EDX spectra were recorded are shown.

For $Ni_{0.7}Pd_{0.2}Bi$, data on a rod-like crystal were collected. The average Ni:Pd:Bi ratio observed is 0.73(2) : 0.19(1) :1, which corresponds to $Ni_{0.73(2)}Pd_{0.19(1)}Bi$, which is within two standard deviations of the single crystal composition.

$Ni_{0.6}Pt_{0.4}Bi$

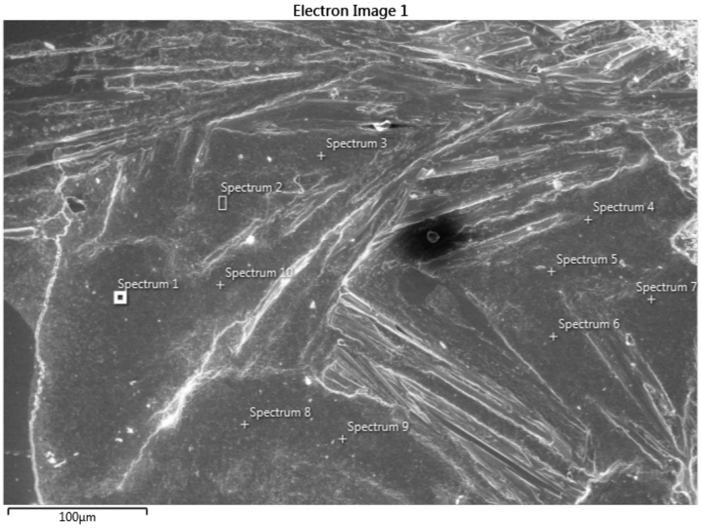

Figure S4. A conglomeration of crystals as a result of the Pt doped NiBi crystal growth as imaged by SEM. Points at which EDX spectra were recorded are shown.

For the Pt doped NiBi system, two distinct compositions were observed, one Ni rich (rod-like grains) and one Pt rich (layer-like grains). The Ni rich rod like grains have an average Ni:Pt ratio of 1.7:1 with a standard deviation of 0.7 – this is a large variance but is consistent with the single crystal composition of an NiAs type phase of $Ni_{0.6}Pt_{0.4}Bi$. The average ratio of Ni:Pt for the Pt rich areas is 0.4:1, with a standard deviation of 0.2. This indicates this phase is likely an Ni doped Pt bismuthide, which is a worthy subject to future study. Accurate determination of Bi content was difficult due to significant remnants of Bi flux.

$Mn_{0.99}Pd_{0.01}Bi$

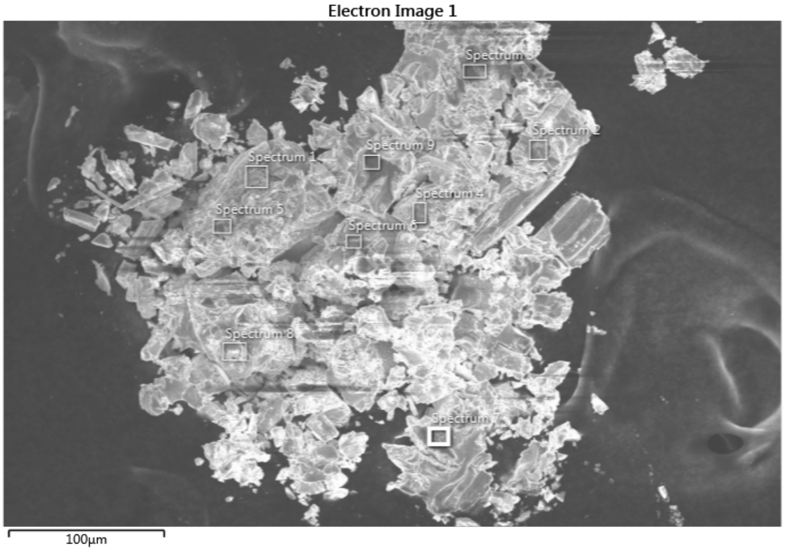

Figure S5. A collection of Pd doped MnBi crystals as imaged by SEM. The Mn,Pd and Bi atomic% are shown in Table S16. Points at which EDX spectra were recorded are shown.
The Pd doped MnBi crystals have varying amounts of Pd, ranging from a Mn:Pd ratio of 1:.007 to 1:.034. The Bi content is usually in excess of 50%, due to remaining Bi flux on the surfaces of the crystals. The average Mn:Pd ratio from the various spots is 1:0.0134 with a standard deviation of 0.0089; as such the composition can be quoted to one significant figure as $Mn_{0.99(1)}Pd_{0.01(1)}Bi$, with the understanding that the Pd content is indeed non-zero.

Table S16. Mn, Bi and Pd atomic percentages at various spots from the collection of crystals as shown in Figure S5.

Mn (%)	Bi (%)	Pd (%)
53.58	45.48	0.94
44.35	55.33	0.31
48.91	50.71	0.38
35.2	64.53	0.27
34.23	65.46	0.32
41.77	57.74	0.49
36.56	62.2	1.23

7 Structural discussion

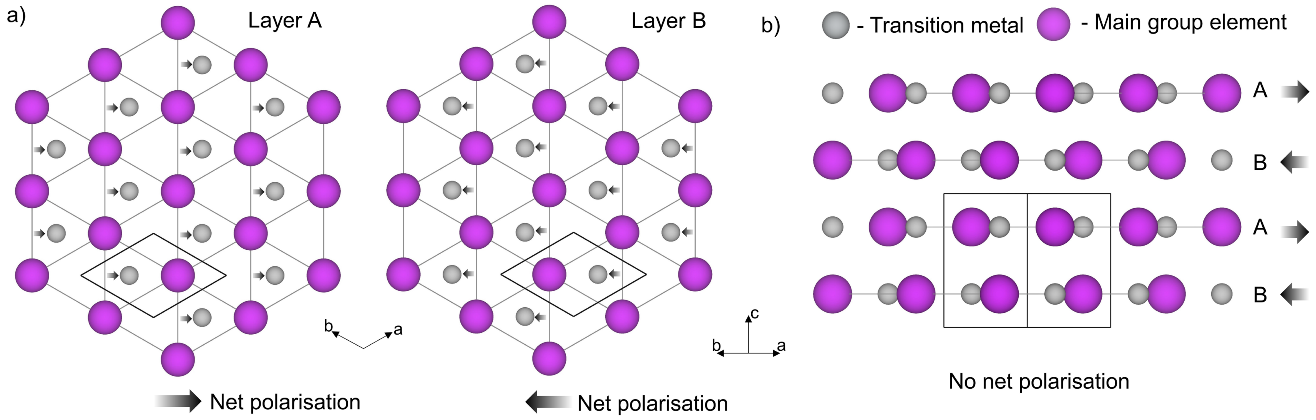

Figure S6. A schematic based on the NiAs structure-type showing how the polar ordering of transition metal interstitials within a hexagonal close-packed main group plane such as in $P6_3/mmc$. The octahedral transition metal atoms are omitted for clarity. a) a hcp lattice of main group elements in which all trigonal interstitial sites are occupied by a transition metal, which generates a net in-plane polarization. The arrows show the displacement of transition metal from the centre of the rhombus formed by the main group element; if the transition metal occupied a site at the centre of each rhombus, the in-plane polarisation would be zero. b) The 6_3 screw axis defines the ABAB stacking of the hcp main group elements and also the transition metal interstitial sites which leads to the cancelling of the polarization of each layer in the kagome-NiAs structure.

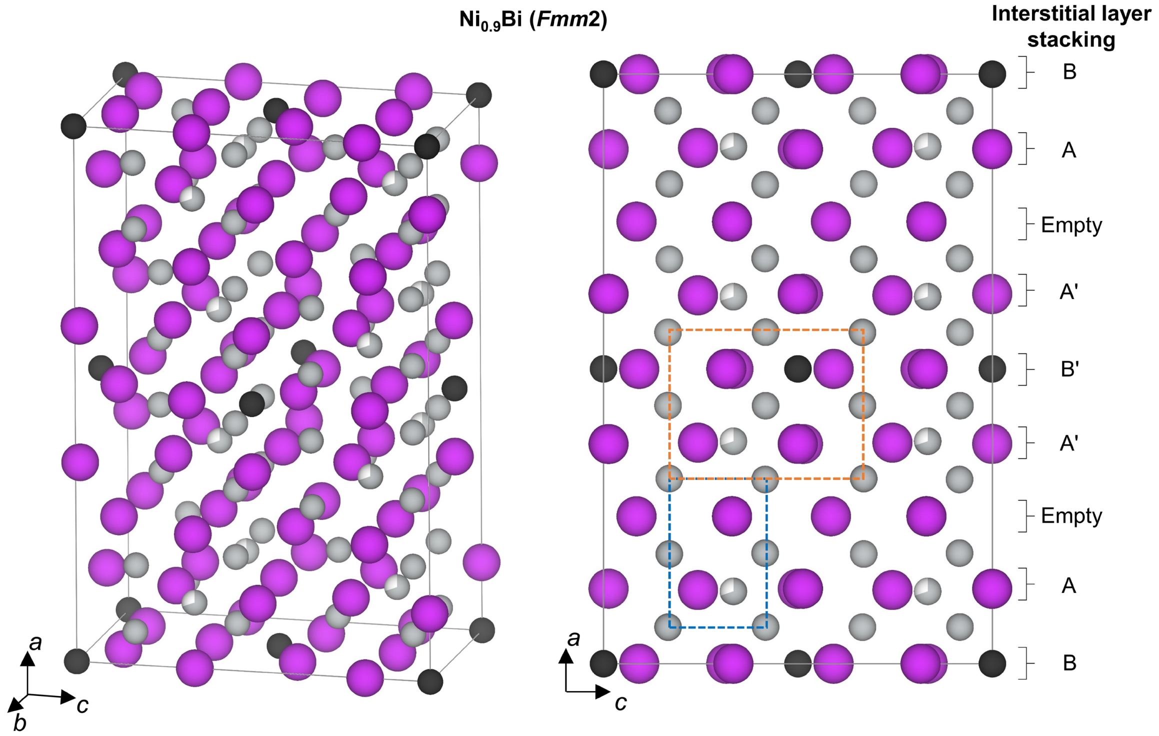

Figure S7. a) Structure of polar kagome-NiAs $Ni_{0.9}Bi$ where the origin has been shifted by 0, 0, 0.3 to demonstrate that the F centring and four-fold expansion of the unit cell along the stacking axis *a* results from the ordering of the trigonal bipyramidal interstitial layers which adopt an A-B-A-empty-A′-B′-A′-empty layer stacking as shown in b). The A′-B′-A′ set of interstitial layers is translated by half a unit cell length along the *c* axis relative to the A-B-A layer set. The Ni site in layer B (4*a* Wyckoff site) is coloured black to emphasise the F centring. The blue dashed outline corresponds to the unit cell of $P6_3/mmc$ NiAs, and the orange dashed outline corresponds to the 2×2×1 expanded unit cell of kagome-NiAs materials reported here ($Ni_{0.6}Pt_{0.4}Bi$, $Ni_{0.7}Pd_{0.2}Bi$, and $Mn_{0.99}Pd_{0.01}Bi$).

In contrast with the structures of $Ni_{0.7}Pd_{0.2}Bi$ and $Ni_{0.6}Pt_{0.4}Bi$, there is a small amount of residual electron density around one of the bismuth sites in $Mn_{0.99}Pd_{0.01}Bi$, which can be modelled as a partially occupied Bi site with low occupancy of 0.035(5). This site is shown in Figure S7. This density was observed in multiple single crystal diffraction measurements at this location, independent of sample and size and geometry, indicating that it is not an artefact resulting from absorption effects. This may hint at some form of layer disorder or local symmetry breaking in $Mn_{0.99}Pd_{0.01}Bi$, and similar observations have been recently observed in Cu-doped MnBi.[8] Full understanding of these interesting features could be provided by examination of local structure via electron microscopy or pair distribution function analysis.

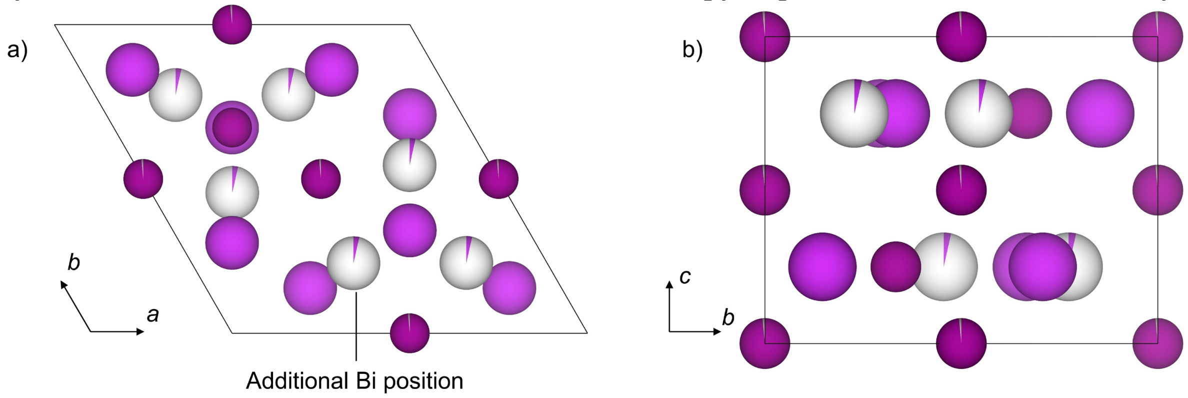

Figure S8. Crystal structure of Kagome-NiAs $Mn_{0.99}Pd_{0.01}Bi$ with 2×2×1 superstructure of NiAs. A small amount of residual electron density near one of the bismuth sites can be modelled as a partially occupied Bi site with low occupancy of 0.035(5). Atom colours: Mn – dark purple; Pd – grey; Bi – light purple.

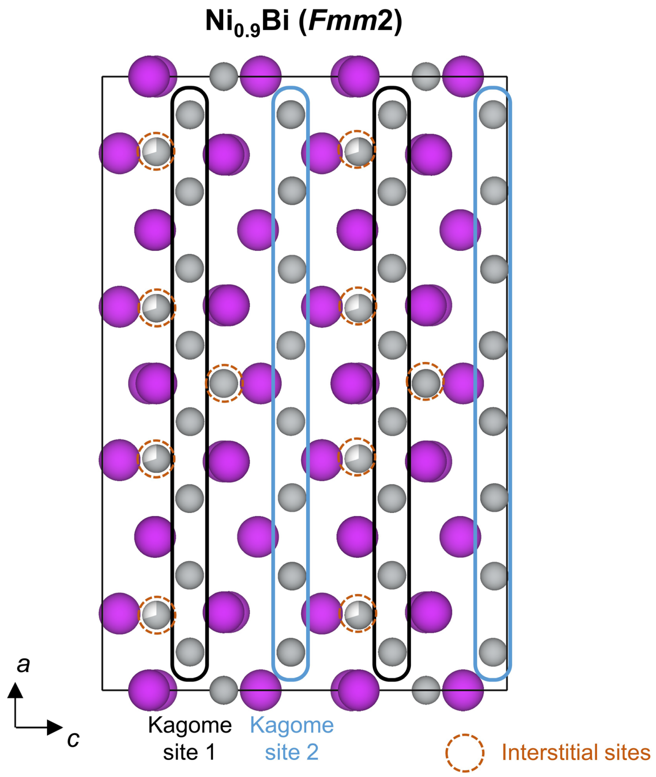

Figure S9. The two distinct octahedral transition metal sites and their proximities to nearby occupied interstitial sites within separate kagome layers in the structures of $Ni_{0.9}Bi$ (left) and $Ni_{0.79}Pd_{0.08}Bi$ (right). The interstitial sites are encircled by orange dashed lines.

8 Energy calculations

The supercells used to calculate the DFT formation energies are shown in Figure S10 and Table S17, in which the symmetry prototype, the space group before and after relaxation, and the energies are provided to reproduce Figure 3(b). All supercells are fully relaxed with the magnitudes of forces on all atoms falling within 0.001 eV/Å, and the electronic self-consistent loops are converged to within 10^{-10} eV.

The convex hull of the DFT formation energies is formed by the $R\overline{3}m$ Bi (s-1), $C2/m$ $NiBi_2$ (s-41 and s-42), and $Fm\overline{3}m$ Ni (s-2). The $C2/m$ $NiBi_2$ was first reported as a high-pressure phase,[9] while both s-41 (high-pressure) and s-42 (ambient-pressure) were relaxed to the same state without constraints on the lattice parameters, see Table S17. The *Fmm*2 $Ni_{0.9375}Bi$ (s-5) and $Ni_{0.90625}Bi$ (s-6) were calculated to be 1.1 meV/atom and 2.5 meV/atom above the convex hull.

Where it was necessary to model partial occupancy of Ni sites in structures, ordered supercells were generated using by CASMcode, which is capable of enumerating symmetrically-distinct structures given the occupations of a specific site and the compositions.[7] For example, for the $P6_3/mmc$-NiAs structure of $Ni_{0.9375}Bi$, three Ni-vacancy supercells (s-8, s-9, and s-10) were generated starting from the four atom NiBi unit cell shown by s-7, see Figure S9 and Table S17. For the Kagome-NiAs structure of $Ni_{0.9375}Bi$, the Ni_8Bi_8 Kagome-NiAs unit cell (s-11) was used, and eight $Ni_{15}Bi_{16}$ structures were generated based on doubled cells (s-12 to s-19). For structural models based on the previously reported Ni_xBi structures with $C2/m$ symmetry, the primitive cell ($Ni_{17}Bi_{16}$, s-20) was generated based on the reported ICSD-410875.[10] The $Ni_{16}Bi_{16}$ supercells (s-20 to s-28) were generated using the s-20. Structure s-22 exhibits the same *Cm* symmetry and energies as s-3, which was reported on Materials Project as mp-1220533.[11] DFT calculations showed that the *Cm* structure is the most stable Ni-vacancy structure within the *C*2/*m* prototype; therefore, s-22 was further used to enumerate the $Ni_{15}Bi_{16}$ supercells (s-29 to s-40).

Introducing Ni-vacancy and structural relaxation may change the space group symmetry of the prototype supercells. The space group symmetries of the input supercells and relaxed supercells were examined using findsym,[12] and are reported under the "Initial SG" and "Final SG" columns in Table S17, respectively. After structural optimization, the symmetries of the supercells remained unchanged except for s-34, which changed from *Cm* symmetry to the *Fmm*2 symmetry. This relaxed s-34 is consistent with s-5 in the same symmetry that the energy difference is 0.1 meV/atom, which is within the convergence uncertainty.

The energies of the different configurations of *C*2/*m* indicate that the polar Kagome-NiAs $Ni_{0.9375}Bi$ can be generated from the $C2/m$ $Ni_{1.0625}Bi$ by producing the Ni-vacancies in an energetically favorable manner, see Figure S12. The most stable Ni-vacancy in *C*2/*m* symmetry resides in Ni-interstitial sites of the Bi layer, leading to the *Cm* symmetry at the composition of NiBi, see s-3 and s-22 in Table S17. The *Fmm*2 structure of $Ni_{0.9375}Bi$ can be stabilized by introducing the Ni-vacancy on the pure-Ni layer adjacent to the pure Bi layer. The resulting *Fmm*2 structure is different from the *C*2/*m* prototype that all Ni-layers in the *Fmm*2 are Kagome nets while two of the Ni-layers in the *C*2/*m* are hexagonal nets, meaning that Ni deficiency is necessary to stabilize the new phase. This supports the experimental observation of the lower Ni:Bi ratio in the *Fmm*2 crystal. The ordering of the Ni-vacancies transforms the hexagonal Ni layers in the *C*2/*m* to Kagome layers while the interstitial Ni content remains largely unchanged.

Entropy contributions are expected in the synthesized compounds provided in Table 1 due to the site occupation disorder. The entropy for disorder sublattice sites in the crystal can be evaluated by the sublattice model:[13]

$$S = -\frac{k_B(\sum_s \alpha^s \sum_i^N x_i^s \ln(x_i^s))}{\sum_s \alpha^s}$$

where k_B is the Boltzmann constant, α^s represents the number of sites on the sublattice-s, x_i^s is the concentration of element-i on the sites of sublattice-s. Table S19 shows the calculated entropy contributions to the energies at 500˚C for all the compounds in Table 1.

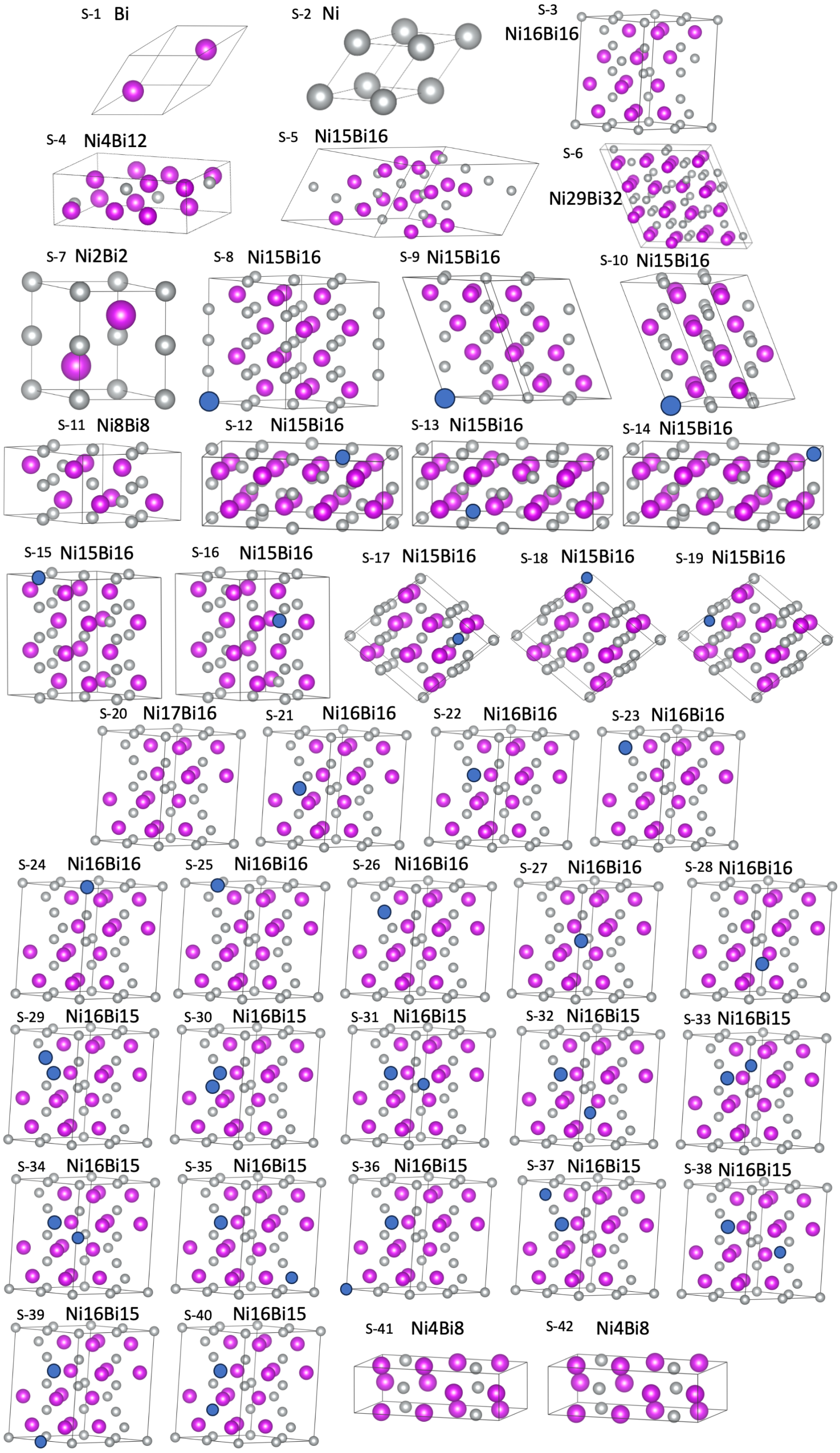

Figure S10. Supercells used in the DFT calculations. The structure indices (s-i) correspond to the structure indices in Table S17. The magenta spheres represent Bi atoms, the silver spheres are Ni atoms, and the Ni-vacancy sites are masked by the blue spheres.

SUPPORTING INFORMATION

Table S17. Structural information and DFT calculated energies of the supercells in the Ni-Bi system. s-1, s-2, s-4, and s-41 marked by bold text form the convex hull in the phase diagram shown in Figure S10.

	symmetry prototype	MPID	ICSD	Formula	Note	y in $Ni_{1-y}Bi_y$	Initial SG	Final SG	DFT Total Energy (eV/atom)	DFT Formation Energy (eV/atom)	DFT Energy above hull (eV/atom)
**1**	$R\bar{3}m$	**mp-23152**	**64703**	**Bi**	**Perfect**	**1**	$R\bar{3}m$	$R\bar{3}m$	**-3.8876**	**0**	0
**2**	$Fm\bar{3}m$	**mp-23**	**37502**	**Ni**	**Perfect**	**0**	$Fm\bar{3}m$	$Fm\bar{3}m$	**-5.4888**	**0**	0
3	*Cm*	mp-1220533	-	$Ni_{16}Bi_{16}$	Perfect	0.5	*Cm*	*Cm*	-4.713	-0.0248	0.0083
**4**	***Pnma***	**mp-23179**	**58821, 391336**	$\mathbf{Ni_4Bi_{12}}$	**Perfect**	**0.75**	***Pnma***	***Pnma***	**-4.3186**	**-0.0307**	0.0024
5	*Fmm2*	NA	-	$Ni_{15}Bi_{16}$	Ni-vacancy	0.516	*Fmm2*	*Fmm2*	-4.6954	-0.033	0.0011
6	*Fmm2*	NA	-	$Ni_{29}Bi_{32}$	Ni-vacancy	0.525	*Fmm2*	*Fmm2*	-4.6811	-0.0322	0.0025
7	*P6₃/mmc-NiAs*	mp-999318	58820, 616868	Ni_2Bi_2	Perfect	0.5	$P6_3/mmc$	$P6_3/mmc$	-4.7069	-0.0187	0.0144
8	$P6_3/mmc$-*NiAs*	NA	58821, 391336	$Ni_{15}Bi_{16}$	Ni-vacancy	0.516	$P\bar{3}m1$	$P\bar{3}m1$	-4.6791	-0.023	0.0101
9	$P6_3/mmc$-*NiAs*	NA	58821, 391336	$Ni_{15}Bi_{16}$	Ni-vacancy	0.516	*C2/m*	*C2/m*	-4.6792	-0.0167	0.0174
10	$P6_3/mmc$-*NiAs*	NA	58821, 391336	$Ni_{15}Bi_{16}$	Ni-vacancy	0.516	*C2/m*	*C2/m*	-4.6787	-0.0168	0.0174
11	*Kagome-NiAs*	NA	-	Ni_8As_8	Perfect	0.5	$P6_3/mmc$	$P6_3/mmc$	-4.7112	-0.0164	0.0178
12	*Kagome-NiAs*	NA	-	$Ni_{15}Bi_{16}$	Ni-vacancy	0.516	$P\bar{1}$	$P\bar{1}$	-4.6789	-0.0165	0.0176
13	*Kagome-NiAs*	NA	-	$Ni_{15}Bi_{16}$	Ni-vacancy	0.516	*Pmm2*	*Pmm2*	-4.6864	-0.024	0.0102
14	*Kagome-NiAs*	NA	-	$Ni_{15}Bi_{16}$	Ni-vacancy	0.516	*P2/m*	*P2/m*	-4.6796	-0.0172	0.0169
15	*Kagome-NiAs*	NA	-	$Ni_{15}Bi_{16}$	Ni-vacancy	0.516	*C2/m*	*C2/m*	-4.6796	-0.0172	0.0169
16	*Kagome-NiAs*	NA	-	$Ni_{15}Bi_{16}$	Ni-vacancy	0.516	$P\bar{6}m2$	$P\bar{6}m2$	-4.6867	-0.0243	0.0099
17	*Kagome-NiAs*	NA	-	$Ni_{15}Bi_{16}$	Ni-vacancy	0.516	$P\bar{1}$	$P\bar{1}$	-4.6794	-0.017	0.0172
18	*Kagome-NiAs*	NA	-	$Ni_{15}Bi_{16}$	Ni-vacancy	0.516	*C2/m*	*C2/m*	-4.6791	-0.0167	0.0175
19	*Kagome-NiAs*	NA	-	$Ni_{15}Bi_{16}$	Ni-vacancy	0.516	*Imm2*	*Imm2*	-4.687	-0.0246	0.0095
20	*C2/m*	NA	410875	$Ni_{17}Bi_{16}$	Perfect	0.485	*C2/m*	*C2/m*	-4.7321	-0.0134	0.0197
21	*C2/m*	NA	410875	$Ni_{16}Bi_{16}$	Ni-vacancy	0.5	*C2*	*C2*	-4.7016	-0.0248	0.0083
22	*C2/m*	NA	410875	$Ni_{16}Bi_{16}$	Ni-vacancy	0.5	*Cm*	*Cm*	-4.713	-0.0177	0.0154
23	*C2/m*	NA	410875	$Ni_{16}Bi_{16}$	Ni-vacancy	0.5	*Cm*	*Cm*	-4.7059	-0.0101	0.023
24	*C2/m*	NA	410875	$Ni_{16}Bi_{16}$	Ni-vacancy	0.5	*C2/m*	*C2/m*	-4.6983	-0.0129	0.0202
25	*C2/m*	NA	410875	$Ni_{16}Bi_{16}$	Ni-vacancy	0.5	$P\bar{1}$	$P\bar{1}$	-4.7011	-0.0157	0.0174
26	*C2/m*	NA	410875	$Ni_{16}Bi_{16}$	Ni-vacancy	0.5	*P1*	*P1*	-4.7039	-0.0244	0.0087
27	*C2/m*	NA	410875	$Ni_{16}Bi_{16}$	Ni-vacancy	0.5	*Cm*	*Cm*	-4.7126	-0.0127	0.0204
28	*C2/m*	NA	410875	$Ni_{16}Bi_{16}$	Ni-vacancy	0.5	*Cm*	*Cm*	-4.701	-0.0196	0.0124
29	*Cm*	NA	410875	$Ni_{15}Bi_{16}$	Ni-vacancy	0.516	*P1*	*P1*	-4.6831	-0.0207	0.0135
30	*Cm*	NA	410875	$Ni_{15}Bi_{16}$	Ni-vacancy	0.516	*P1*	*P1*	-4.6829	-0.0205	0.0136
31	*Cm*	NA	410875	$Ni_{15}Bi_{16}$	Ni-vacancy	0.516	*Cm*	*Cm*	-4.6827	-0.0203	0.0138
32	*Cm*	NA	410875	$Ni_{15}Bi_{16}$	Ni-vacancy	0.516	*Cm*	*Cm*	-4.68	-0.0176	0.0166
33	*Cm*	NA	410875	$Ni_{15}Bi_{16}$	Ni-vacancy	0.516	*Cm*	*Cm*	-4.6795	-0.0172	0.017
34	*Cm*	NA	410875	$Ni_{15}Bi_{16}$	Ni-vacancy	0.516	*Cm*	*Fmm2*	-4.6953	-0.0329	0.0012
35	*Cm*	NA	410875	$Ni_{15}Bi_{16}$	Ni-vacancy	0.516	*Cm*	*Cm*	-4.6833	-0.0209	0.0133
36	*Cm*	NA	410875	$Ni_{15}Bi_{16}$	Ni-vacancy	0.516	*Cm*	*Cm*	-4.6767	-0.0143	0.0199
37	*Cm*	NA	410875	$Ni_{15}Bi_{16}$	Ni-vacancy	0.516	*Cm*	*Cm*	-4.684	-0.0216	0.0125
38	*Cm*	NA	410875	$Ni_{15}Bi_{16}$	Ni-vacancy	0.516	*C2/m*	*C2/m*	-4.6947	-0.0323	0.0018
39	*Cm*	NA	410875	$Ni_{15}Bi_{16}$	Ni-vacancy	0.516	*P1*	*P1*	-4.6793	-0.017	0.0172
40	*Cm*	NA	410875	$Ni_{15}Bi_{16}$	Ni-vacancy	0.516	*P1*	*P1*	-4.6832	-0.0208	0.0133
**41**	***C2/m***	**NA**	**-**	$\mathbf{Ni_4Bi_8}$	**Perfect**	**0.667**	***C2/m***	***C2/m***	**-4.4655**	**-0.0441**	**0**
**42**	***C2/m***	**NA**	**-**	$\mathbf{Ni_4Bi_8}$	**Perfect**	**0.667**	***C2/m***	***C2/m***	**-4.4655**	**-0.0441**	**0**

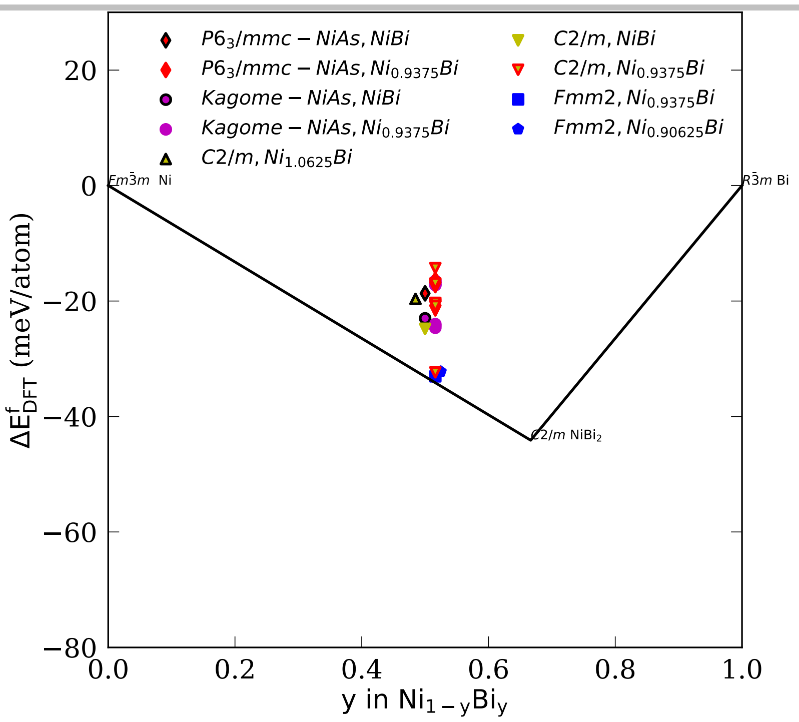

Figure S11. DFT formation energies of all the structures reported in Table S17 and Figure S10.

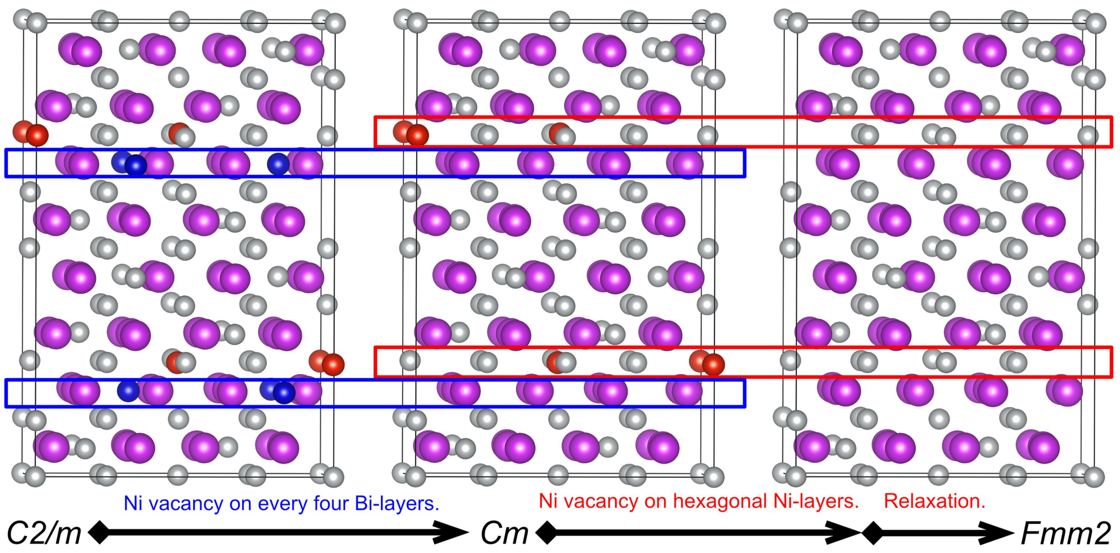

Figure S12. Schematic illustration of stabilizing *Fmm2* $Ni_{0.9375}Bi$ by introducing and ordering of the Ni-vacancies in the *C2/m* structure (s-20). First, the *Cm* structure NiBi (s-3 and s-22) can be generated by producing Ni vacancies (blue atoms in the left structure) on every four Bi layers in the *C2/m* prototype (s-20). Second, by producing Ni vacancies (red atoms in the middle structure) in the hexagonal Ni planes of the *Cm* structure, the new structure can be stabilized as the *Fmm*2 phase through subsequent relaxation (s-5 and s-34). Atom colors: grey – Ni, purple – Bi, blue - Ni-vacancy on Bi-layer, and red - Ni-vacancy on Ni hexagonal layer.

Table S18. Entropy contributions to the energies of the synthesized compounds.

Material	Symmetry	Structure	S (meV/atom/K)	ST at 500˚C (meV/atom)
$Ni_{0.6}Pt_{0.4}Bi$	*P6₃/mmc*	"kagome-NiAs"	0.0223	17.269
$Ni_{0.7}Pd_{0.2}Bi$	*P6₃/mmc*	"kagome-NiAs"	0.0222	17.162
$Mn_{0.99}Pd_{0.01}Bi$	*P6₃/mmc*	"kagome-NiAs"	0.0072	5.560
$Ni_{0.9}Bi$	*Fmm*2	"Polar kagome-NiAs"	0.0034	2.634
$Ni_{0.79}Pd_{0.08}Bi$	*Fmm*2	"Polar kagome-NiAs"	0.0064	4.934

9 Magnetisation measurements

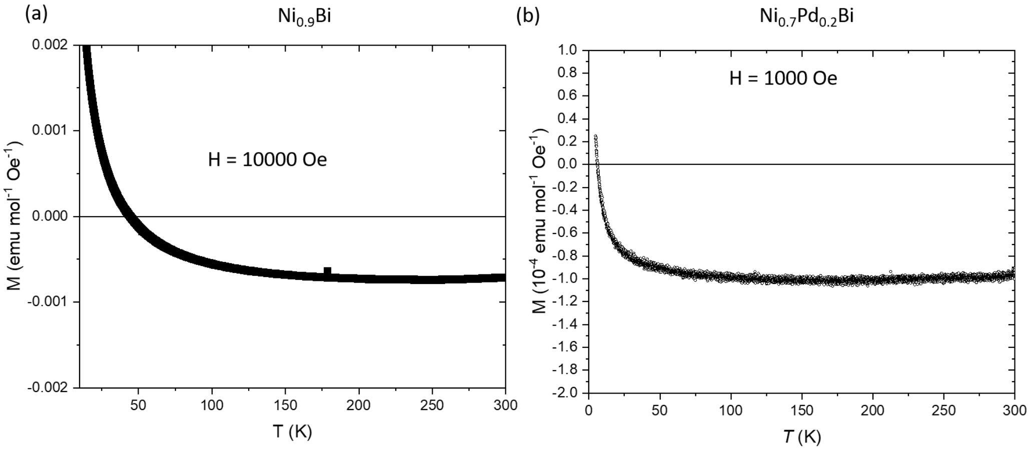

Figure S13. (a) Magnetisation of $Ni_{0.9}Bi$ with the external magnetic field of 10000 Oe applied along the *a*-axis (stacking axis). (b) Magnetisation of $Ni_{0.7}Pd_{0.2}Bi$ with the external magnetic field of 1000 Oe applied along the *c*-axis (stacking axis).

The magnetisation of $Ni_{0.9}Bi$ and $Ni_{0.7}Pd_{0.2}Bi$ are shown in Figure S13. Both show a small Curie tail and a diamagnetism at high temperatures which indicate a large orbital diamagnetism.

10 Electronic transport measurements

The Bloch-Gruneisen function we employed here to fit resistivities of $Ni_{0.7}Pd_{0.2}Bi$ is modified with an additional Einstein term:

$$\rho(T) = \rho_0 + A\left(\frac{T}{T_B}\right)^n \int_0^{\frac{T_B}{T}} \frac{t^n}{(e^t-1)(1-e^{-t})}\mathrm{d}t + \frac{B}{T(e^{\frac{T_E}{T}}-1)(1-e^{\frac{-T_E}{T}})},$$

where T_B is the Bloch-temperature temperature and T_E is the Einstein temperature, and the parameters A and B are the relative weights.

Samples of $Ni_{0.9}Bi$ and $Ni_{0.7}Pd_{0.2}Bi$ show a superconducting transition at 4 K, likely due to a small impurity of the known superconductor $NiBi_3$. Heat capacity (Figure S14) of $Ni_{0.9}Bi$ and $Ni_{0.7}Pd_{0.2}Bi$ samples show no presence of superconductivity, consistent with the interpretation of impurity superconductivity from small amounts of $NiBi_3$. We suggest that previous reports of superconductivity in NiAs-type NiBi may be due to contamination with $NiBi_3$.[9, 14]

11 Heat Capacity

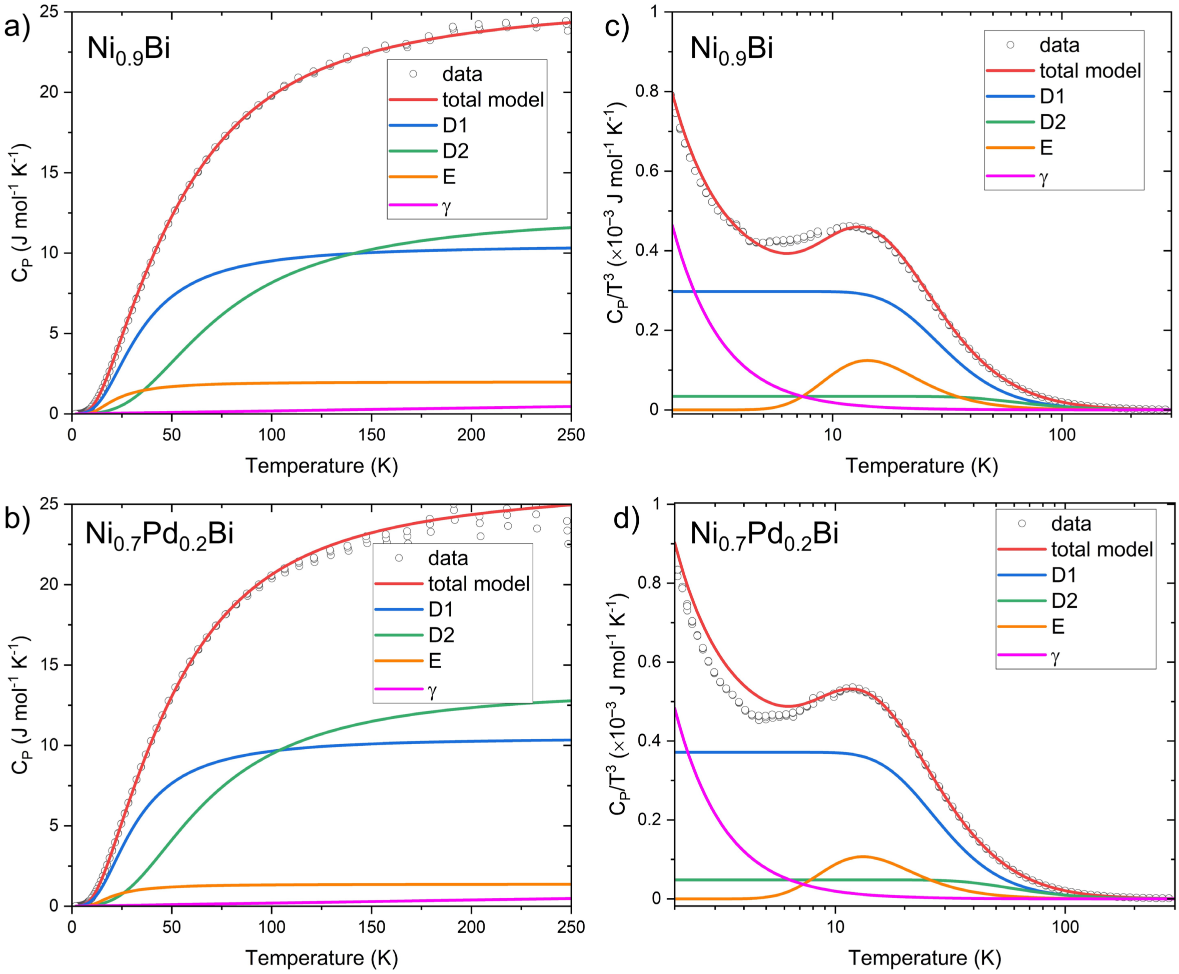

Figure S14. Heat capacity for (a) $Ni_{0.9}Bi$ and (b) $Ni_{0.7}Pd_{0.2}Bi$, and plotted as C_P/T^3 for (c) $Ni_{0.9}Bi$ and (d) $Ni_{0.7}Pd_{0.2}Bi$. The relative contribution of the two Debye terms (D1 and D2), the Einstein term (E) and the electronic term (γ) are shown.

The heat capacities of $Ni_{0.9}Bi$ and $Ni_{0.7}Pd_{0.2}Bi$ are shown in Figure S14 and both fit with two Debye terms, one Einstein term and one linear term from the electronic contribution. The fitting function is:

$$C_p = C_p^{D1} + C_p^{D2} + C_p^{E} + C_e$$

$$= 9 \cdot R \left[a \cdot \left(\frac{T}{\Theta_{D1}}^{3}\right) \int_0^{\Theta_{D1}} \frac{x^4 e^x}{(e^x - 1)^2} dx + b \cdot \left(\frac{T}{\Theta_{D2}}^{3}\right) \int_0^{\Theta_{D2}} \frac{x^4 e^x}{(e^x - 1)^2} dx \right] + \frac{3R \cdot r \left(\frac{\Theta_E}{T}\right)^2 \cdot \exp\left(\frac{\Theta_E}{T}\right)}{\left(\exp\left(\frac{\Theta_E}{T}\right) - 1\right)} + \gamma T,$$

where Θ_{D1} and Θ_{D2} are the Debye temperatures, Θ_E is the Einstein temperature, γ is the linear electronic term, and the parameters a, b, r are the relative weights. In order to keep the high temperature limit to 3NR, the coefficients a, b and r were constrained such that a+b+r = 1.

The low-temperature and high-temperature data can be both fit well by this function. Besides, it can be seen that no first-order transitions are observed for both materials, suggesting the absence of structural transitions in the measured temperature range.

12 References

[1] O. V. Dolomanov, L. J. Bourhis, R. J. Gildea, J. A. K. Howard, H. Puschmann, *J. Appl. Crystallogr.* **2009**, *42*, 339-341.

[2] G. Sheldrick, *Acta Crystallogr. Sect. A* **2015**, *71*, 3-8.

[3] G. Sheldrick, *Acta Crystallogr. Sect. C* **2015**, *71*, 3-8.

[4] P. E. Blöchl, *Phys. Rev. B* **1994**, *50*, 17953-17979.

[5] G. Kresse, J. Furthmüller, *Phys. Rev. B* **1996**, *54*, 11169-11186.

[6] J. P. Perdew, K. Burke, M. Ernzerhof, *Phys. Rev. Lett.* **1996**, *77*, 3865-3868.

[7] B. Puchala, J. C. Thomas, A. R. Natarajan, J. G. Goiri, S. S. Behara, J. L. Kaufman, A. Van der Ven, *Comput. Mater. Sci.* **2023**, *217*, 111897.

[8] G. Angelo, J. G. Philbrick, J. Zhang, T. Kong, X. Gui, *arXiv:2308.08952 [cond-mat.mtrl-sci]* **2023**, https://doi.org/10.48550/arXiv.42308.08952.

[9] S. M. Clarke, K. M. Powderly, J. P. S. Walsh, T. Yu, Y. Wang, Y. Meng, S. D. Jacobsen, D. E. Freedman, *Chem. Mater.* **2019**, *31*, 955-959.

[10] M. Ruck, *Z. Anorg. Allg. Chem.* **1999**, *625*, 2050-2054.

[11] A. Jain, S. P. Ong, G. Hautier, W. Chen, W. D. Richards, S. Dacek, S. Cholia, D. Gunter, D. Skinner, G. Ceder, K. A. Persson, *APL Mater.* **2013**, *1*, 011002.

[12] H. T. Stokes, D. M. Hatch, *J. Appl. Crystallogr.* **2005**, *38*, 237-238.

[13] M. Hillert, *Phase Equilibria, Phase Diagrams and Phase Transformations: Their Thermodynamic Basis*, 2 ed., Cambridge University Press, Cambridge, **2007**.

[14] B. Silva, R. F. Luccas, N. M. Nemes, J. Hanko, M. R. Osorio, P. Kulkarni, F. Mompean, M. García-Hernández, M. A. Ramos, S. Vieira, H. Suderow, *Phys. Rev. B* **2013**, *88*, 184508.
